# A two-stage genome-wide association study to identify novel genetic loci associated with acute radiotherapy toxicity in nasopharyngeal carcinoma

**DOI:** 10.1186/s12943-022-01631-8

**Published:** 2022-08-23

**Authors:** Yang Wang, Fan Xiao, Yi Zhao, Chen-Xue Mao, Lu-Lu Yu, Lei-Yun Wang, Qi Xiao, Rong Liu, Xi Li, Howard L. McLeod, Bi-Wen Hu, Yu-Ling Huang, Qiao-Li Lv, Xiao-Xue Xie, Wei-Hua Huang, Wei Zhang, Cheng-Xian Guo, Jin-Gao Li, Ji-Ye Yin

**Affiliations:** 1 Department of Clinical Pharmacology, Xiangya Hospital, Central South University, Changsha, 410078 P. R. China; 2 Institute of Clinical Pharmacology, Central South University, Hunan Key Laboratory of Pharmacogenetics, Changsha, 410078 P. R. China; 3 Engineering Research Center of Applied Technology of Pharmacogenomics, Ministry of Education, 110 Xiangya Road, Changsha, 410078 P. R. China; 4 National Clinical Research Center for Geriatric Disorders, 87 Xiangya Road, Changsha, 410008 Hunan P.R. China; 5 Department of General Practice, Shenzhen People’s Hospital (The Second Clinical Medical College, Jinan University, The First Affiliated Hospital, Southern University of Science and Technology), Shenzhen, 518020 Guangdong P.R. China; 6 Geriatric Oncology Consortium, Tampa, FL 33612 USA; 7 USF Taneja College of Pharmacy, Tampa, FL 33612 USA; 8 Center of Clinical Pharmacology, the Third Xiangya Hospital, Central South University, Changsha, 410013 Hunan P. R. China; 9 Department of Radiation Oncology, Jiangxi Cancer Hospital of Nanchang University, Nanchang, 330029 P.R. China; 10 National Health Commission (NHC) Key Laboratory of Personalized Diagnosis and Treatment of Nasopharyngeal Carcinoma (Jiangxi Cancer Hospital of Nanchang University), Nanchang, 330029 P.R. China; 11 Departent of Radiotherapy, Hunan Provincial Tumor Hospital and Affiliated Tumor Hospital of Xiangya Medical School, Central South University, Changsha, 410013 P.R. China; 12 Hunan Key Laboratory of Precise Diagnosis and Treatment of Gastrointestinal Tumor, Changsha, 410078 P. R. China

**Keywords:** Nasopharyngeal carcinoma, GWAS, Radiogenomics, Radiotherapy toxicity, Skin reaction, Dysphagia, INHBB, STY8

## Abstract

**Background:**

Genetic variants associated with acute side effects of radiotherapy in nasopharyngeal carcinoma (NPC) remain largely unknown.

**Methods:**

We performed a two-stage genome-wide association analysis including a total of 1084 patients, where 319 individuals in the discovery stage were genotyped for 688,783 SNPs using whole genome-wide screening microarray. Significant variants were then validated in an independent cohort of 765 patients using the MassARRAY system. Gene mapping, linkage disequilibrium, genome-wide association analysis, and polygenic risk score were conducted or calculated using FUMA, LDBlockShow, PLINK, and PRSice software programs, respectively.

**Results:**

Five SNPs (rs6711678, rs4848597, rs4848598, rs2091255, and rs584547) showed statistical significance after validation. Radiotherapy toxicity was more serious in mutant minor allele carriers of all five SNPs. Stratified analysis further indicated that rs6711678, rs4848597, rs4848598, and rs2091255 correlated with skin toxicity in patients of EBV positive, late stage (III and IV), receiving both concurrent chemoradiotherapy and induction/adjuvant chemotherapy, and with OR values ranging from 1.92 to 2.66. For rs584547, high occurrence of dysphagia was found in A allele carriers in both the discovery (*P* = 1.27 × 10^− 6^, OR = 1.55) and validation (*P* = 0.002, OR = 4.20) cohorts. Furthermore, prediction models integrating both genetic and clinical factors for skin reaction and dysphagia were established. The area under curve (AUC) value of receiver operating characteristic (ROC) curves were 0.657 (skin reaction) and 0.788 (dysphagia).

**Conclusions:**

Rs6711678, rs4848597, rs4848598, and rs2091255 on chromosome 2q14.2 and rs584547 were found to be novel risk loci for skin toxicity and dysphagia in NPC patients receiving radiotherapy.

**Trial registration:**

Chinese Clinical Trial Register (registration number: ChiCTR-OPC-14005257 and CTXY-140007-2).

**Supplementary Information:**

The online version contains supplementary material available at 10.1186/s12943-022-01631-8.

## Introduction

Nasopharyngeal carcinoma (NPC) is an epithelial malignant tumor arising from the lining of nasopharynx [1]. The incidence of NPC has a unique geographical distribution pattern, with Chinese patients accounting for approximately 50% of all patients. In China, NPC mainly occurs in southern regions, including Hunan, Jiangxi, Guangxi, Guangdong, and Fujian provinces [2]. The main treatment methods for NPC include radiotherapy, chemotherapy, and immunotherapy. Among these, radiotherapy has an important role. However, side effects, which can be divided into acute and late toxicity according to the occurrence of time point, are common and serious. Late toxicity is generally a toxic reaction occurring within 1 to 3 years after the end of radiotherapy, while acute toxicity occurs within 3 months. Almost half of the patients reportedly suffer acute grade 2–3 toxicities, including oral mucositis, skin reaction, dysphagia, neutropenia, etc. [3]. Although some toxicities could be relieved after supportive care measures, they could be associated with lingering morbidity or even death [4]. Thus, it is important to discover the contributors of acute radiotherapy toxicity for NPC patients and explore their prediction performance in the clinic.

Radiogenomics aims to identify genetic variants contributing to radiotherapy effects and toxicity [3]. Over the past decade, the genome-wide association study (GWAS) has emerged as one of the major strategies for radiogenomic investigation and has successfully identified some genetic variants associated with radiation toxicity in cancers of the prostate, breast, cervix, and lung [3, 5–7]. For NPC, radiogenomic studies employing GWAS to identify susceptibility loci for radiotherapy toxicity are still limited. Some genetic variants were found to be involved in the development of neurotoxicity and oral mucositis [8, 9]. For other acute toxicities, a study used as the main method sample candidate gene association in a small sample size. Genetic variants associated with radiation-induced dysphagia, skin reaction salivary gland toxicity, and myelosuppression still largely remain unknown.

In this study, we performed a two-stage GWAS study using a total of 1084 NPC patients that aimed to discover the genetic variants associated with radiotherapy acute toxicity and evaluate their clinic prediction performance.

## Methods

### Study population and data collection

All patients in this study were recruited from Jiangxi Province Tumor Hospital (Nanchang, Jiangxi, China) and Hunan Cancer Hospital (Changsha, Hunan, China), from October 2014 to December 2017. Patients were diagnosed with NPC by histopathological examination and treated by intensity modulated radiation therapy (IMRT) with 6-MV photons at a dose of 2 Gy/d, 5 times a week. The prescribed doses were calculated according to the gross tumor volume (GTV), clinical target volume (CTV), and planning target volume (PTV). Primary lesions and positive cervical lymph nodes received 66–77 Gy and 54–60 Gy in 30–33 fractions, respectively. A platinum-based regimen was used as induction, adjuvant, or concurrent chemoradiotherapy. Patients did not undergo surgery, targeted therapy, immunotherapy, or other anti-tumor therapy before radiotherapy. Patients with serious concomitant diseases that might greatly affect their physical condition were excluded.

A physical examination as well as a detailed inquiry into each patient’s medical history was carried out. Acute radiotherapy toxicities were evaluated based on criterion of the Radiation Therapy Oncology Group or European Organization for Research and Treatment of Cancer (RTOG/EORTC) [10]. Based on this scoring criteria, grade 0 was absent of toxicities, while grade 5 signified the death of patients. Grade 1–4 represented increasing severity of toxicities. In the present study, skin reaction, dysphagia, oral mucositis, salivary gland toxicity, and myelosuppression were evaluated. For the association analysis, all patients were further divided into two groups: mild (grade 0 and 1) and moderate/severe toxicity (grade 2 and 3), since no grade 4 and 5 toxicities were found. It should be noted that patients were divided into two groups based on the presence or absence of toxicity for myelosuppression, since few patients had moderate or severe toxicities (grades 2–5). Therapeutic responses of all patients were assessed according to response evaluation criteria in solid tumors (RECIST) 1.1 version [11]. Patients with a complete response (CR) or a partial response (PR) were defined as responders. Patients showing stable disease (SD) or progressive disease (PD) were defined as non-responders.

**Fig. 1:**
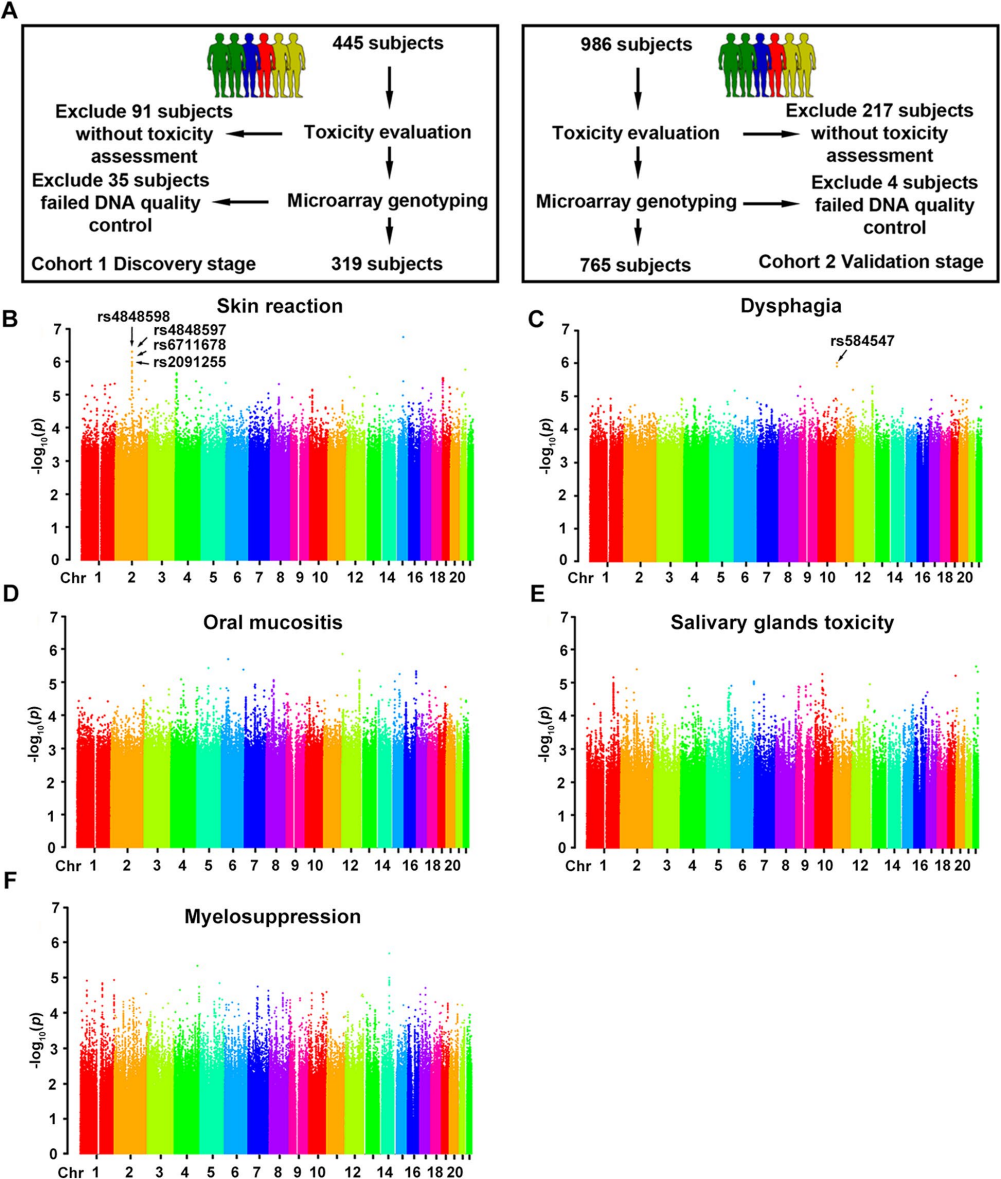
Genome-wide association study design and results in discovery cohort. **A** Flow diagram of clinical study design. **B**-**E** Manhattan plot showed the genome-wide association results for skin reaction (**A**), dysphagia (**B**), oral mucositis (**C**), salivary gland toxicity (**D**), and myelosuppression (**E**). SNPs successfully validated in the validation cohort are indicated. Association *P* values are expressed as -log_10_(p). *P* values were calculated from multivariate logistic regression analysis

The study protocol was approved by the Ethics Committee of Jiangxi Province Tumor Hospital and Hunan Cancer Hospital. All patient information and data from both hospitals were collected using the same protocol, and there was no overlap of patients. All subjects were provided with written informed consents. We applied this study for clinical admission in the Chinese Clinical Trial Register (registration number: ChiCTR-OPC-14005257). As indicated in Fig. 1A, this was a two-stage GWAS study. A total of 1426 patients were initially enrolled, 345 patients were excluded due to absence of toxicity assessment, failed DNA quality control, or failed genotyping quality control. Ultimately, 1084 patients were included in the two-stage analysis.

### Genotyping, quality control, and whole genome imputation

The detailed process of GWAS analysis, bioinformatic tools utilized, and their parameters in this study are summarized in Fig. S1. In brief, genotyping was performed using the Global Screening Array-24 v1.0 BeadChip (Illumina, San Diego, CA, USA) for 688,783 SNPs. Genotype calls were determined using GenomeStudio software from Illumina (San Diego, CA, USA). The systematic quality control was performed on the raw genotyping data to filter both unqualified samples and SNPs using PLINK (1.9 version). After sample relatedness, heterozygosity and sex check, samples were excluded if they failed genotyping in more than 10% of variants. SNPs were excluded if: (1) SNPs were not mapped on autosomal chromosomes; (2) SNPs had a call rate < 95%; (3) SNPs had MAF < 0.05; or (4) SNPs deviated significantly from Hardy-Weinberg equilibrium (HWE) (*P* < 1 × 10^− 5^). IMPUTE2 was used to perform phasing and imputation on the filtered un-phased genotypes to impute un-typed SNPs based on 1000 Genomes Phase 3 v4 reference [12]. Imputed genotypes with IMPUTE2 info lower than 0.4 were discarded for association analysis and SNPs that had MAF < 0.05 were further excluded. Finally, a total of 4,112,760 unique SNPs were included in the GWAS analysis. In the validation stage, SNP genotyping was performed using the Sequenom MassARRAY system (Sequenom, San Diego, CA, USA) according to the manufacturer’s instructions. The exclusion criteria included: (1) After sample relatedness, heterozygosity and sex check, samples that failed genotyping in more than 10% of variants; (2) SNPs that had a call rate < 90%; (3) SNPs that had MAF < 0.05; or (4) SNPs that deviated significantly from Hardy-Weinberg equilibrium (HWE).

### Genome-wide association analysis

In the discover stage, principal component analysis (PCA) was performed using data from all patients using PLINK (1.9 version) program (Fig. S2) [13]. The genomic inflation factor of population stratification λ was also calculated by PLINK (1.9 version). The chi square test was employed to evaluate the association between clinical factors and various toxicities. The significantly associated factors were considered covariates. For five phenotypes of skin reaction--dysphagia, oral mucositis, salivary gland toxicity, and myelosuppression--GWAS was conducted under the additive, dominant, and recessive models by multivariate logistic regression analysis, adjusted by age, sex, body mass index (BMI), smoking status, stage, Epstein-Barr virus (EBV) infection, and radiotherapeutic regimen as covariates.

### Bioinformatics and statistical analysis

The linkage disequilibrium (LD) and haplotype block estimation analyses were performed using PLINK (1.9 version) and LDBlockShow (1.35 version). Regional plots and gene mapping were generated using the online tool LocusZoom (http://locuszoom.sph.umich.edu/) and functional mapping and annotation (FUMA) v1.3.4c (https://fuma.ctglab.nl/) [14, 15]. Transcription regulation was predicted based on the data of ENCODE database (https://www.encodeproject.org/) [16]. The polygenic risk score was calculated and evaluated by PRSice-2 v2.3.5 [17]. All other statistical analyses were performed using SPSS 20.0 software (SPSS Inc., Chicago, Illinois, USA). In this study, all *P-*values were two-sided and *P* ≤ 0.05 was considered to be statistically significant.

## Results

### Clinical characteristics

This two-stage genome-wide association study involved a total of 1084 subjects. Among them, a total of 319 patients from Jiangxi Province Tumor Hospital were recruited into the discovery stage cohort, and 765 patients from Hunan Cancer Hospital were used to validate the results. Table 1 summarizes the demographic characteristics of all patients. The whole-genome microarray analysis was performed on peripheral blood DNA samples from 319 patients in the discovery stage. After quality control and genotype imputation, a total of 4,112,760 SNPs passed quality screening and were included in the association analysis.

**Table 1:** Characteristics of all patients

Characteristics	Discovery stage	Validation stage
Number of patients	319	765
Age (Mean ± SD)	50.75 ± 11.96	48.30 ± 9.87
Gender		
Male	215	570
Female	104	195
BMI (Mean ± SD)	22.61 ± 3.40	23.16 ± 3.30
Smoking status		
Smoker	149	372
Non-smoker	166	370
NR	4	23
Clinical stage		
I	7	19
II	26	64
III	120	325
IV	165	307
NR	1	50
EBV		
Positive	216	408
Negative	103	311
NR	0	46
Treatment scheme		
RT alone	60	12
RT + IC/AC	4	0
CCRT	100	26
CCRT+IC/AC	154	606
RT + other treatment^a^	1	121

*SD* Standard deviation, *BMI* Body mass index, *EBV* Epstein-Barr virus, *NR* Not reported, *RT* Radiotherapy, *IC* Induction chemotherapy, *AC* Adjuvant chemotherapy, *CCRT* Concurrent chemoradiotherapy

^a^Other treatment included surgery, targeted therapy, immunotherapy and herbs treatment

In the discovery stage of this study, five acute radiotherapy toxicities of skin reaction, dysphagia, oral mucositis, salivary gland toxicity, and myelosuppression were investigated. The characteristics of NPC patients involved in these association analyses are summarized in Tables S1, S2, S3, S4 and S5. For each phenotype, the association of clinical characteristics of age, gender, BMI, smoking status, EBV infection, clinical stage, and treatment scheme between different subgroups was analyzed. All significant clinical factors were considered as covariates and adjusted in subsequent GWAS analysis.

### GWAS analysis for five toxicities

For GWAS analysis, a quantile-quantile plot was created to evaluate the data quality (Fig. S3). Deviation from the expected *P* value distribution was evident only in the tail region. For the results of the association analysis, the genomic inflation factor λ was 0.971, 1.017, 0.989, 0.976, and 0.981 for skin reaction, dysphagia, oral mucositis, salivary gland toxicity, and myelosuppression, respectively. In addition, PCA results showed that no stray samples appeared in any toxicity (Fig. S2). The results also indicated that the patient population was ethnically homogeneous, and the observed associations were not driven by potential population substructure. Thus, no principal components (PC) were incorporated as covariates in the following association analysis. The Manhattan plot in Fig. 1B-E shows the results of five toxicities in discovery stage. Variations with *P* < 1 × 10^− 5^ (dysphagia, oral mucositis, salivary gland toxicity, and myelosuppression) or *P* < 1 × 10^–5.5^ (skin reaction) were selected for MassArray genotyping in the validation stage samples. As indicated in Table 2, a total of 16 SNPs were finally successfully genotyped. For myelosuppression, no SNPs satisfied the quality control criteria in the validation cohort, rendering this phenotype incompletely assessed. For oral mucositis and salivary gland toxicity, no SNPs were successfully replicated as being associated with the specific toxicity in the validation cohort. Finally, five SNPs were successfully validated by showing statistical significance.

**Table 2:** The SNPs associated with acute radiotherapy toxicity in nasopharyngeal carcinoma patients

CHR	SNPs	Gene	Allele	MAF	Discovery stage		Validation stage	
					*P* value	OR	*P* value	OR
Skin reaction								
2	rs6711678	INHBB	G/C	0.28	7.17 × 10^−7^	1.23	0.009	1.46
2	rs4848597	INHBB	C/T	0.30	4.82 × 10^−7^	1.34	0.014	1.42
2	rs4848598	INHBB	G/A	0.28	4.91 × 10^−7^	1.36	0.010	1.45
2	rs2091255	INHBB	G/T	0.24	9.83 × 10^−7^	1.40	0.007	1.48
Dysphagia								
5	rs670902	MGAT1	T/C	0.40	6.71 × 10^−6^	1.98	0.656	0.75
9	rs149372542	None	G/A	0.18	5.13 × 10^−6^	1.03	0.250	1.82
11	rs584547	SYT8	G/A	0.27	1.27 × 10^− 6^	1.55	0.002	4.20
11	rs111570505	None	A/G	0.19	1.41 × 10^−6^	0.40	0.212	1.86
12	rs14143	BRI3BP	G/A	0.49	5.18 × 10^−6^	0.99	0.521	1.36
Oral mucositis								
5	rs10214299	EDIL3	A/C	0.47	3.79 × 10^−6^	0.93	0.984	1.00
6	rs1334970	SLC25A27	T/C	0.30	2.04 × 10^−6^	1.45	0.524	1.12
12	rs4766031	TSPAN9	G/A	0.42	1.41 × 10^−6^	0.96	0.662	0.94
6	rs11754905	SLC25A27	C/T	0.31	2.00 × 10^−6^	0.69	0.705	1.07
Salivary glands toxicity								
10	rs73328964	ASAH2	T/C	0.05	5.51 × 10^−6^	1.65	0.719	1.16
10	rs73328981	ASAH2	C/T	0.05	5.51 × 10^−6^	1.13	0.515	1.51
22	rs35806646	RASD2	T/C	0.14	3.28 × 10^−6^	1.35	0.719	1.16

Rs6711678, rs4848597, rs4848598, and rs2091255 correlated with skin reaction. These four SNPs were all located on the chromosome 2q14.2 and showed high LD with each other. They showed similar *P* and OR value in both the discovery (*P* value ranged from 4.82 × 10^− 7^ to 9.83 × 10^− 7^, OR value ranged from 1.23 to 1.40) and validation cohort (*P* value ranged from 0.007 to 0.014, OR value ranged from 1.42 to 1.48) for the minor allele. In addition, rs584547 was identified to be associated with high occurrence of dysphagia for A allele in both the discovery (*P* = 1.27 × 10^− 6^, OR = 1.55) and validation (*P* = 0.002, OR = 4.20) cohort. Thus, these five SNPs underwent further analysis.

### Stratified and meta-analysis of five associated variants

As previously reported, clinical factors may also contribute to drug or radiotherapy toxicities [18, 19]. Therefore, we conducted stratified analysis for these five SNPs in the combined cohort. Clinical characteristics only showed significant influence on the genetic correlation of rs6711678, rs4848597, rs4848598, and rs2091255. Thus, these SNPs were further analyzed. As indicated in Table S6, for smoking status, the association existed in both smokers and non-smokers. However, for EBV infection, clinical stage, and treatment scheme, the association only existed in subgroups of EBV positive, late stage (III and IV), and patients receiving both concurrent chemoradiotherapy and induction/adjuvant chemotherapy. This result indicated that clinical factors might affect the results of radiotherapy toxicity analysis. For example, half of EBV positive patients who were homozygous mutant for these four SNPs also experienced grade 2 or 3 skin toxicity after receiving radiotherapy. In contrast, this ratio was less than 25% in the wild-type patients.

**Fig. 2:**
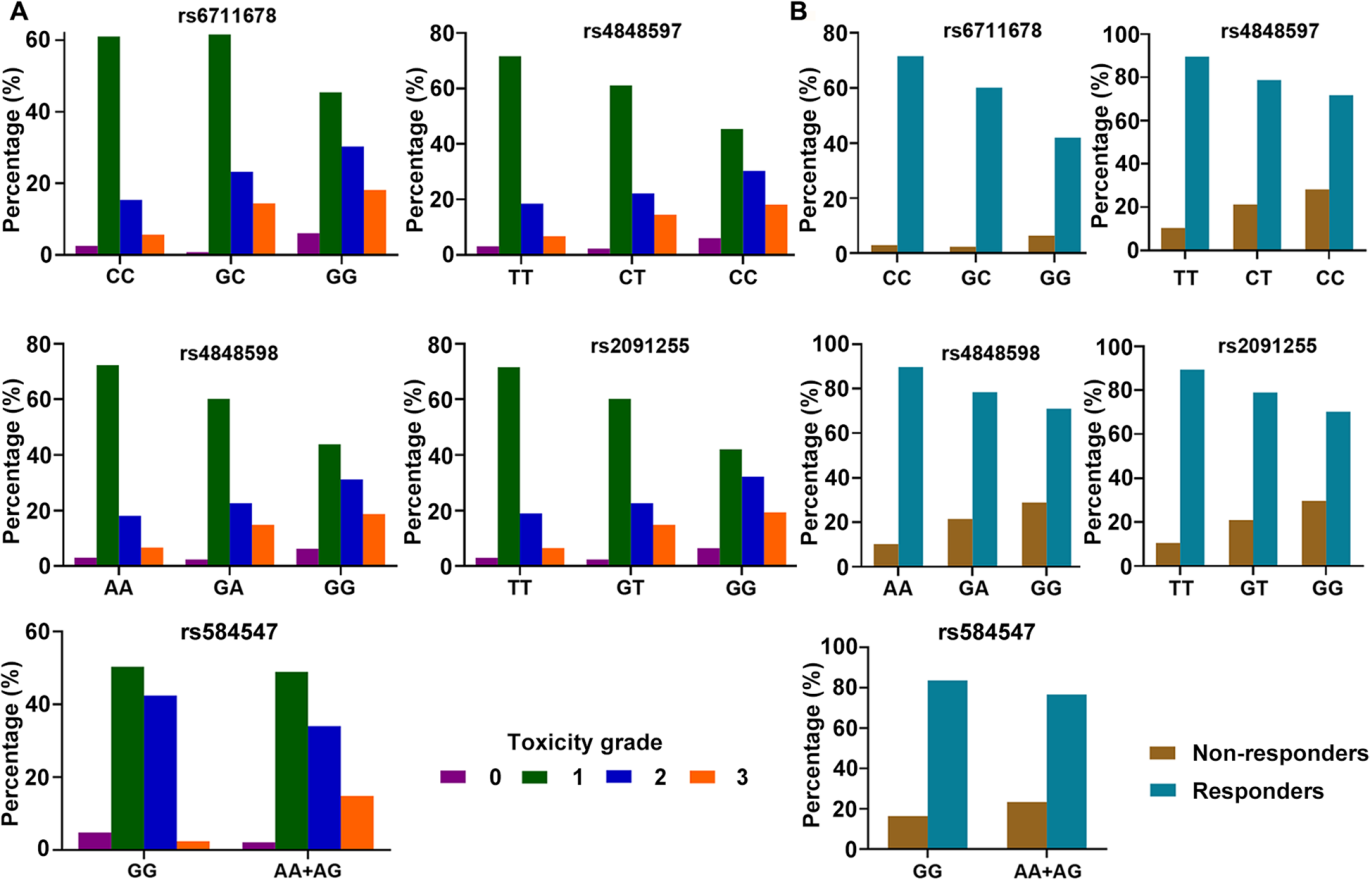
Patient distribution in different genotypes according to skin reaction toxicity (**A**) and radiotherapeutic response (**B**). For 2q14.2 loci, all patients were EBV positive, late stage (III and IV), and receiving both concurrent chemoradiotherapy and induction/adjuvant chemotherapy. For rs584547, AA genotypes were combined in analysis with AG heterozygote patients due to the limited number

Based on these findings, we considered that rs6711678, rs4848597, rs4848598, and rs2091255 were more associated with skin reaction in NPC patients who were EBV positive, late stage (III and IV), and receiving both concurrent chemoradiotherapy and induction/adjuvant chemotherapy. We thus further conducted the association analysis in this subgroup. As indicated in Table 3, the results are consistent in three different models for all SNPs. Minor allele carriers showed higher risk of skin reaction with an OR value increasing to more than 2. Figure 2 supports this result more intuitively by calculating the patients with different grades of toxicities in groups of three genotypes. For all SNPs, occurrence of grade 2 and 3 skin reaction toxicities remarkably increased in minor allele carriers. In contrast, mild toxicity (grade 1) dramatically decreased. We next performed therapeutic response analysis in these patients and observed that there were more non-responders in patients with minor alleles for all four SNPs, with an OR value ranging from 1.92 to 2.66 (Table S7).

**Table 3:** Association between skin reaction and chromosome 2q14.2 loci in the stratified patients

SNP	MAF	Additive		Dominant		Recessive	
		*P* value	OR (95%CI)	*P* value	OR (95%CI)	*P* value	OR (95%CI)
rs6711678	0.30	1.15 × 10^−3^	1.79 (1.26–2.55)	4.54 × 10^−3^	2.00 (1.24–3.22)	0.03	2.24 (1.08–4.64)
rs4848597	0.31	1.93 × 10^−3^	1.74 (1.22–2.47)	7.21 × 10^−3^	1.92 (1.19–3.09)	0.03	2.22 (1.07–4.59)
rs4848598	0.30	6.32 × 10^−4^	1.84 (1.30–2.63)	2.85 × 10^− 3^	2.07 (1.28–3.33)	0.02	2.37 (1.13–4.95)
rs2091255	0.29	7.16 × 10^−4^	1.83 (1.29–2.61)	3.89 × 10^−3^	2.01 (1.25–3.22)	0.02	2.48 (1.18–5.24)

For rs584547, no clinical characteristics were found to significantly influence the genetic correlation. Thus, it was analyzed in the combined cohort without any stratification. Due to the limited number, AA genotypes were combined in analysis with AG heterozygote patients. As indicated in Fig. 2, grade 3 toxicity occurred more in the A allele carriers, while all other grade toxicities were decreased. Higher resistance to the radiotherapy was also found in these patients.

### Toxicity prediction models

**Fig. 3:**
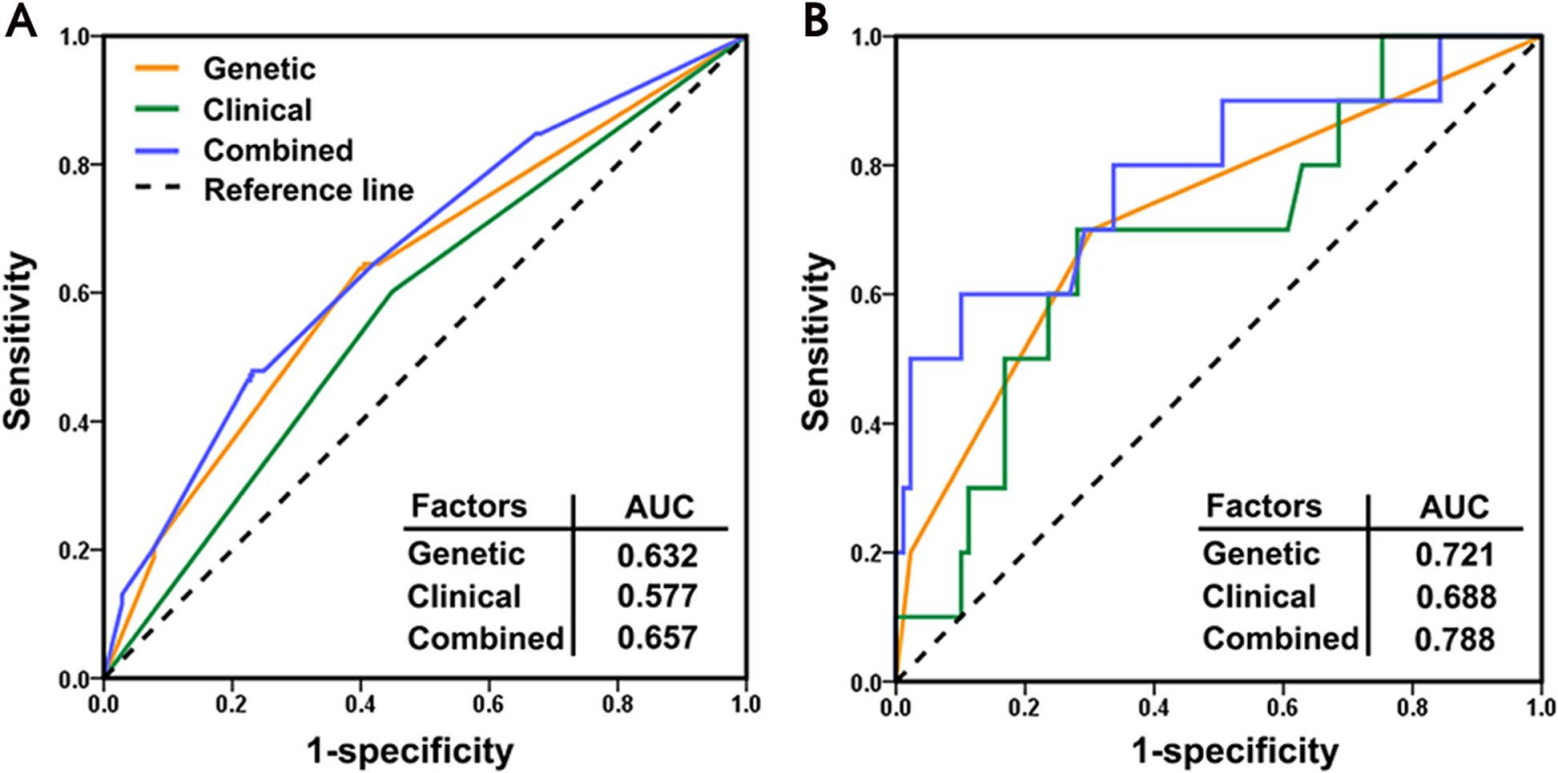
Receiver operating characteristic curves of three models for skin reaction (**A**) and dysphagia (**B**). All models were generated by using multivariable logistic regression. The genetic model only involved genetic factors: rs6711678, rs4848597, rs4848598, and rs2091255 for skin reaction, and rs584547 for dysphagia. During the calculation, rs6711678, rs4848597, rs4848598, and rs2091255 were combined as polygenic risk scores. The clinical model involved clinical factors only, which include age, sex, BMI, smoking status, stage, EBV infection, and radiotherapeutic regimen. The combined model integrated both genetic and clinical factors. BMI: body mass index, EBV: Epstein-Barr virus, AUC: area under curve

Based on above analysis, the toxicity was increased, and response was decreased in minor allele carriers for all five SNPs, which could be considered as potential biomarkers for precision radiotherapy of NPC patients. We thus evaluated their toxicity prediction performance. For skin reaction toxicity and dysphagia, the combined cohort patients were first randomly divided into two groups, which were used to establish and test models, respectively. The sample size of each group accounted for 50% of all patients. Three multivariable logistic regression models were established: with genetic factors only, clinical factors only, and a combination of both genetic and clinical factors. For skin reaction toxicity, four SNPs of rs6711678, rs4848597, rs4848598, and rs2091255 were combined as polygenic risk scores in the genetic-only model after controlling clinical parameters. As indicated in Fig. S4, the area under curve (AUC) value of receiver operating characteristic (ROC) curves of the genetic-only model was larger than that of clinical-only model in both groups for all phenotypes. This result suggested that the genetic model integrating four SNPs was more accurate in classifying skin reaction occurrence patients compared with estimating using only clinical characteristics. After further integrating clinical factors, the ROC AUC of the combined model further increased. The similar performance of each model in both establishing and testing groups supported the reliability of our models. Therefore, we established the final prediction models for two toxicities in the combined cohort. As indicated in Fig. 3A, the ROC AUCs of genetic only, clinical only, and combined factors for skin reaction toxicity were 0.632, 0.577, and 0.657, respectively. The performance was better for dysphagia, where the corresponding values were 0.721, 0.688, and 0.788, respectively (Fig. 3B). These results together showed that these five genetic variants could potentially be used to predict skin reaction and dysphagia toxicities in NPC patients receiving radiotherapy.

### Gene mapping

**Fig. 4:**
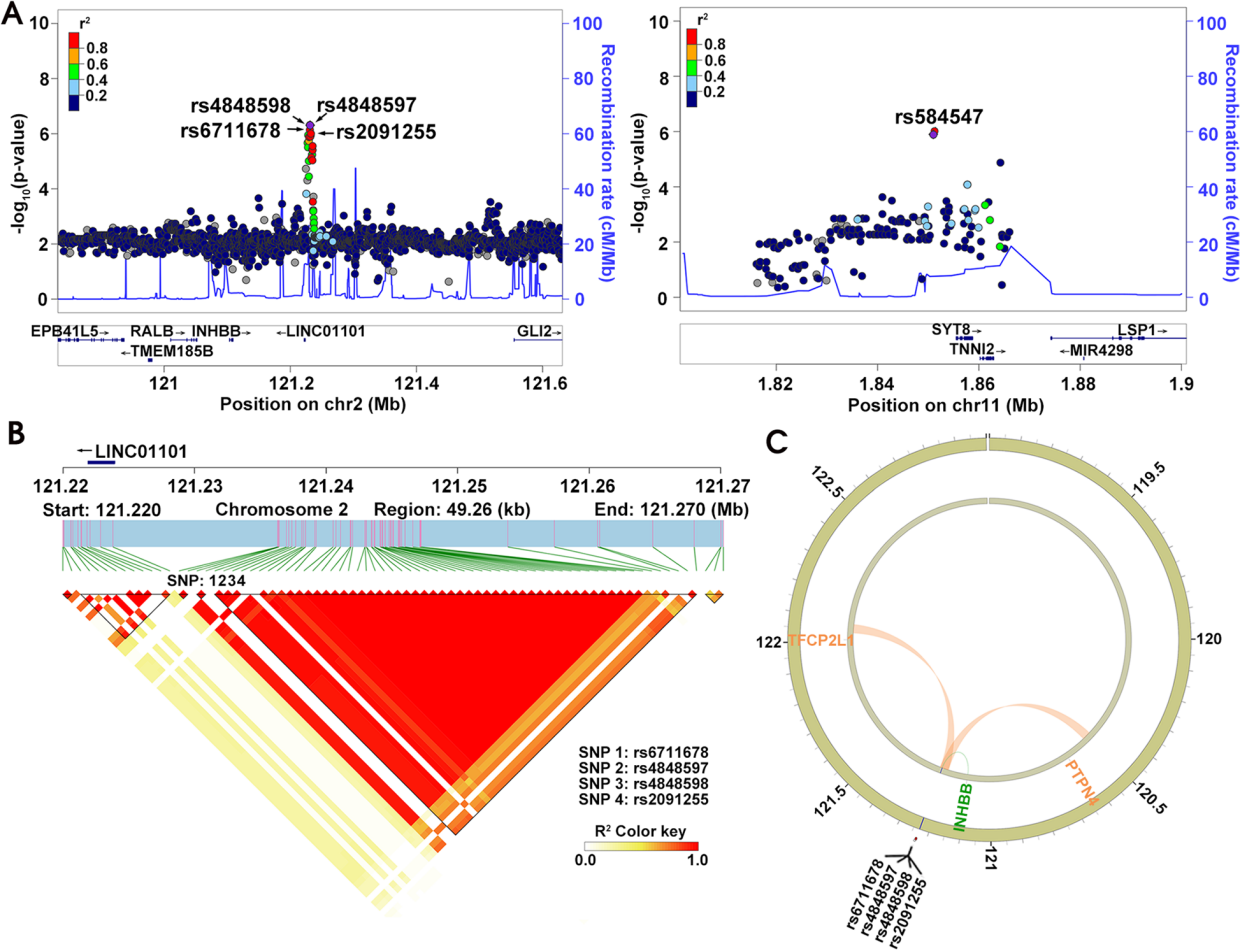
Gene mapping and LD analysis of rs6711678, rs4848597, rs4848598, rs2091255, and rs584547. **A** The regional plot for rs6711678, rs4848597, rs4848598, rs2091255, and rs584547. The regional plots were constructed using LocusZoom. *P* values (−log_10_(*P* values); y axis) were plotted against the respective chromosomal position of each SNP (x axis). Colors indicate LD (r^2^) with four SNPs in 1000 Genomes Project East Asian populations. **B** The haplotype block of four SNPs in chromosome 2. The genomic positions were indicated. **C** 3D chromatin interaction and eQTL analysis of rs6711678, rs4848597, rs4848598, and rs2091255. Circos plot showing genes on chromosome 2 interacted with four SNPs by 3D chromatin interaction (orange line) and eQTL mapping (green line). The outer layer showed the –log_10_(*P* values) of four SNP in the GWAS analysis

To learn the functional consequences of the five risk loci, we mapped them to genes. As indicated in Fig. 4, rs6711678, rs4848597, rs4848598, and rs2091255 were closely located on the chromosome 2q14.2, spanning only 1690 bp. Further analysis showed that all four SNPs were in strong LD in the genome. Although they were closer to a long non-coding RNA (LncRNA) of LINC01101, eQTL analysis implicated genes of inhibin subunit beta B (INHBB). In addition, 3D chromatin interaction indicated that they interacted with transcription factor CP2 like 1 (TFCP2L1) and protein tyrosine phosphatase non-receptor type 4 (PTPN4).

The SNP rs584547 localized very closely upstream of synaptotagmin-8 (SYT8). However, LD analysis showed that it was not in linkage with any proximal SNPs (Fig. 4D). 3D chromatin interaction and eQTL analysis also showed no implicated genes. Considering 5′ upstream SNPs may affect gene expression by altering promoter activity, we evaluated its transcription regulation function using ENCODE database. The result suggested that rs584547 showed a proximal transcriptional regulation function on SYT8 gene.

## Discussion

In the current study, we explored the genetic variants associated with radiotherapy acute toxicity of skin reaction, dysphagia, oral mucositis, salivary gland toxicity, and myelosuppression, using GWAS in a total of 1084 NPC patients. Chromosome 2q14.2 loci (rs6711678, rs4848597, rs4848598, and rs2091255) and rs584547 were found to be significantly associated with skin reaction and dysphagia. The toxicities were more serious in minor allele carriers. Further stratified analysis showed that chromosome 2q14.2 loci correlated with skin reaction in patients that were EBV positive, late stage (III and IV), and receiving both concurrent chemoradiotherapy and induction/adjuvant chemotherapy. The area under curve (AUC) values of receiver operating characteristic (ROC) curves of prediction models integrating both genetic and clinical factors for skin reaction and dysphagia were 0.657 and 0.788, respectively. The mapped genes were INHBB, TFCP2L1, and PTPN4 for 2q14.2 loci, and SYT8 for rs584547.

Radiotherapy is the main treatment method for NPC. However, severe acute side effects often occur in the pharynx, mucous membranes, skin, or hematopoietic system and significantly affect the therapeutic results and quality of life. Previous reports observed that some genetic variants correlated with these toxicities, including X-ray repair cross complementing 1 (XRCC1), XRCC3, XRCC6, transforming growth factor beta 1 (TGFB1), etc. [20, 21]. However, these studies mainly used candidate gene association strategy, with a focus on DNA repair genes. More genetic variants remain to be discovered. Since GWAS provides a powerful strategy to discover novel genetic variants, we conducted this current study to identify novel genetic variants associated with acute radiotherapy toxicity in NPC patients. After validating in an independent cohort from another hospital, we finally found that chromosome 2q14.2 loci (rs6711678, rs4848597, rs4848598, and rs2091255) and rs584547 were associated with skin reaction and dysphagia. These loci were novel and had not been reported in either NPC or other cancers. Previous studies have identified some loci associated with similar radiation-induced toxicities, including rs35542, rs117157809, and NBN (nibrin) variants [22, 23]. However, these loci did not show significant correlation in our study. We speculated that the main reasons were different phenotypes and population. Considering that we discovered these SNPs in the Chinese patients, we explored their ethnic differences by comparing the MAF in six major populations. As indicated in Fig. S5, all five SNPs showed significant ethnic differences. This could be one of the potential reasons of inconsistence results between our studies and others.

**Fig. 5:**
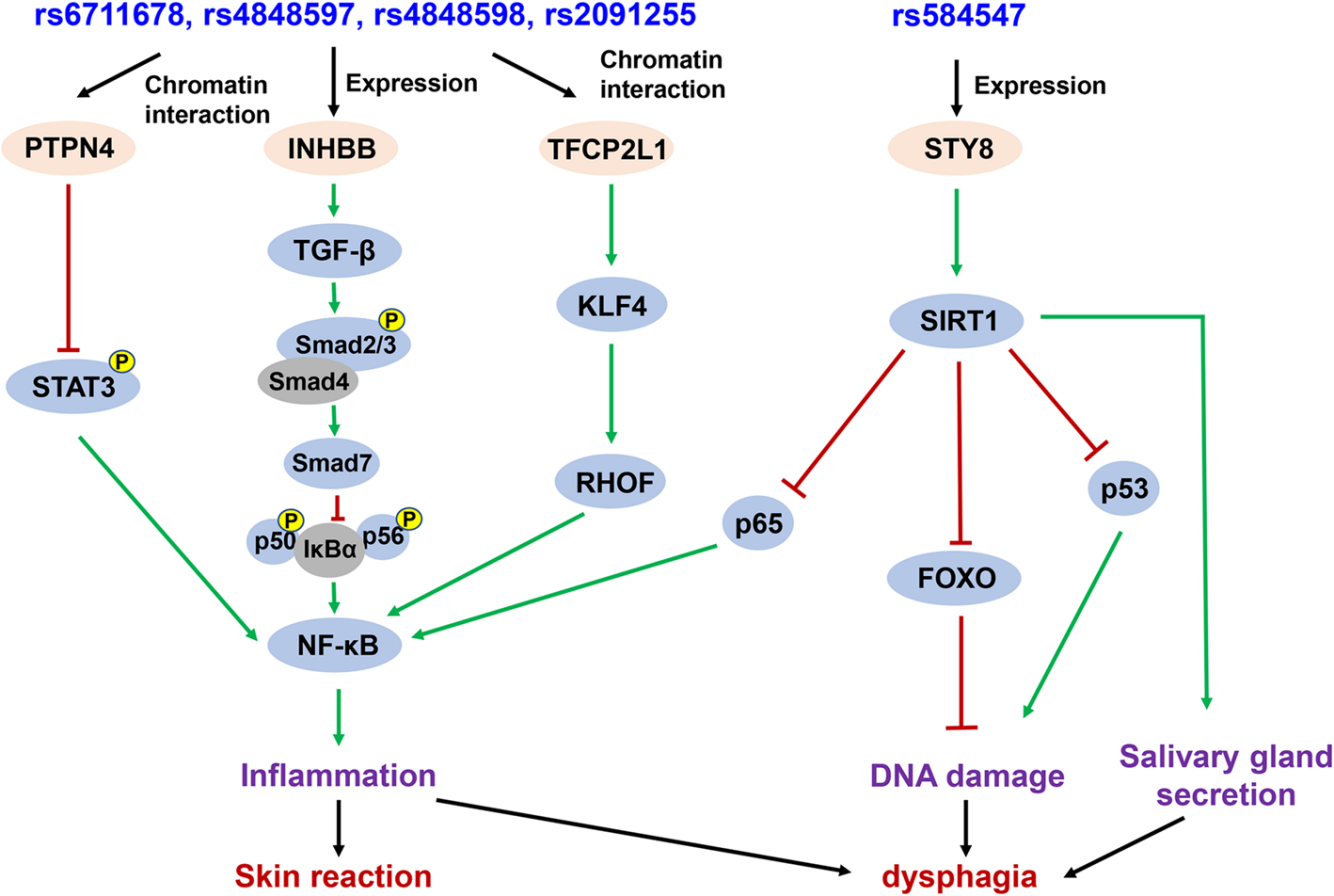
The potential molecular mechanisms of correlation between five susceptibility SNPs and toxicities. Rs6711678, rs4848597, rs4848598, and rs2091255 implicated expression of INHBB, which regulates TGF-β/Smad/IκBα/NF-κB axis. In addition, 3D chromatin interaction indicated that they interacted with TFCP2L1 and PTPN4. PTPN4 belongs to the PTP enzyme family, which hydrolytically removes phosphate groups from proteins. PTPN4 has been reported to be a signaling molecule of STAT3. TFCP2L1 is a transcription regulating factor and reported to play key roles in regulating KLF4/ RHOF/NF-κB pathway. Finally, all three targeted proteins regulate NF-κB, which is a key modulator of inflammation, and correlate with skin reaction toxicity. Rs584547 was very closely located upstream of SYT8 and showed a proximal transcriptional regulation function on this gene. It was reported that SYT8 inhibited p63, FOXO, and p53 by regulating SIRT1, which in turn affected cellular processes of inflammation, DNA damage, and salivary gland secretion. SYT8 may mediate those processes to affect dysphagia. INHBB: inhibin subunit beta B, TGF-β: transforming growth factor-beta, Smad: SMAD family member 2, IκBα: NF-κB inhibitor alpha, NF-κB: nuclear factor kappa B, STAT3: signal transducer and activator of transcription 3, SYT8: synaptotagmin 8, TFCP2L1: transcription factor CP2 like 1, PTPN4: protein tyrosine phosphatase non-receptor type 4, KLF4: KLF transcription factor 4, RHOF: ras homolog family member F, FOXO: forkhead box O, SIRT1:sirtuin 1

Skin reaction is a common toxicity of radiotherapy in both NPC and other cancers; therefore, it attracts great attention. Several studies reported the correlation of genetic variants and skin reaction. However, these studies mainly investigated breast cancer patients using candidate gene association study strategies [24–28]. Polymorphisms of XRCC1 were the most widely investigated. The results contradicted each other [29]. For NPC, little is known about the genetic variants that correlate with skin toxicities. We found that four novel genetic loci on chromosome 2q14.2 correlated with susceptibility of radiotherapy-induced skin reaction. A gene mapping study indicated the implicated genes were INHBB, TFCP2L1, and PTPN4. INHBB encodes a member of transforming growth factor-beta (TGF-β) superfamily proteins. It was reported to suppress anoikis resistance and migration in NPC [30]. As indicated in Fig. 5, it is a key modulator of inflammation by regulating TGF-β/Smad/nuclear factor kappa B (NF-κB) axis. PTPN4 belongs to PTPs enzyme family, which hydrolytically removes phosphate groups from proteins. It was reported that PTPN4 was a signaling molecule of several important pathways, including signal transducer and activator of transcription 3 (STAT3), toll like receptor 4 (TLR4), mitogen-activated protein kinase 1 (MAPK1)*,* etc. [31–34]. TFCP2L1 is a transcription regulating factor and is reported to play key roles in inflammation by regulating KLF transcription factor 4 (KLF4)/ras homolog family member F (RHOF)/NF-κB pathway [35]. Although there is no evidence that shows that INHBB, TFCP2L1, and PTPN4 are directly involved in skin reaction, their regulated signaling pathways and cellular processes are widely reported to be correlated with inflammation as mentioned above [36, 37]. We speculate that this is the possible mechanisms of 2q14.2 loci mutation correlated with patients’ skin reaction.

Dysphagia is also an important common toxicity of radiotherapy in NPC patients. Previously, no genetic variants were reported to be correlated with this toxicity. We identified that rs584547 was a risk variant of dysphagia for the first time. Gene mapping analysis showed that this SNP may regulate SYT8 transcription. It is reasonable to speculate that it may exert function by affecting SYT8 gene expression [38]. This gene encodes a member of the synaptotagmin protein family, which is important in neurotransmission. A recent whole-exome sequencing analysis discovered that a SNP in this gene is correlated with irritable bowel syndrome and depressive disorder in the Chinese population [39]. It was also reported that SYT8 inhibited p63, forkhead box O3 (FOXO), and p53 by regulating sirtuin 1 (SIRT1), which in turn affected cellular processes of inflammation, salivary gland secretion, and damaged DNA [40, 41] (Fig. 5). It is possible that SYT8 mediated those processes and neurotransmissions to affect dysphagia. Thus, the rs584547 mutation regulates SYT8 expression and radiotherapy induced dysphagia.

Radiogenomics is committed to finding genetic biomarkers for prediction of radiotherapeutic effectiveness and safety. In our study, all five loci were newly discovered. This result provided more evidence to support that GWAS is a powerful tool to discover novel radiogenomic biomarkers. To explore the potential ability of these SNPs in clinic toxicity prediction, we established multivariable logistic regression models integrating both genetic and clinical factors. The ROC AUCs reached 0.657 and 0.788 for skin reaction and dysphagia, respectively. These variants substantially improved the accuracy of models. Although their performance still needs to be improved due to the limited contribution of five SNPs, we speculate that formulating a more accurate predictive model incorporating more genetic variants could be implemented in the clinic.

Some limitations should be noted while interpreting our data. First, both our genetic variant and prediction models warrant replication in larger and other cohorts. Second, the ORs associated with all five SNPs ranges from 1.74 to 4.20, indicating that contribution of these variants to the toxicities has limits. More genetic or other factors may be waiting to be discovered. Finally, the detailed mechanisms of five SNPs correlated with toxicity still need to be clarified in vitro. It is difficult to mimic skin reaction and dysphagia toxicity in cell and animal models. These studies were absent in the current study.

## Conclusion

We conducted a two-stage GWAS study to identify genetic variants associated with five major radiotherapy acute toxicities (skin reaction, dysphagia, oral mucositis, salivary gland toxicity, and myelosuppression) in NPC patients. We found that rs6711678, rs4848597, rs4848598, and rs2091255 on chromosome 2q14.2 and rs584547 were novel risk loci for skin reaction and dysphagia. They could be used to optimize the treatment strategies to reduce the acute toxicities in NPC patients.

## Supplementary Information

**Additional file 1: Fig. S1.** Diagram of data processing flow. Bioinformatics tools utilized in each step were showed in blue in the brackets. Detailed parameters and quality control criteria were indicated with red. **Fig. S2.** Distribution of samples according to PCA analysis in discovery stage. The red and green spots represented two different groups of patients. The results showed that no stray samples appeared in all five toxicities. **Fig. S3.** Quantile–quantile (QQ) plot of observed association *P* values (y-axis) against expected *P* values (x-axis) in the discovery stage. **Fig. S4.** Establishment of prediction models for skin reaction (A and B) and dysphagia toxicities (C and D). For each toxicity, patients were firstly randomly divided into two groups, which used to establish (A and C) and test models (B and D) respectively. Then, three multivariable logistic regression models with genetic factors only, clinical factors only and combination of both genetic and clinical factors were established. The genetic model only involved genetic factors: rs6711678, rs4848597, rs4848598 and rs2091255 for skin reaction, and rs584547 for dysphagia. During the calculation, rs6711678, rs4848597, rs4848598 and rs2091255 were combined as polygenic risk scores. The clinical model involved clinical factors only, which include age, sex, BMI, smoking status, stage, EBV infection and radiotherapeutic regimen. The combined model integrated both genetic and clinical factors. BMI: body mass index, EBV: Epstein-Barr virus, AUC: area under curve. **Fig. S5.** The MAF of rs6711678, rs4848597, rs4848598, rs2091255 and rs584547 in different ethnic populations. AFR: African, EAS: East Asian, EUR: Europe, AMR: American, SAS: South Asian, LAM: Latin American. **Table S1.** Characteristics of NPC patients involved in skin reaction association analysis. **Table S2.** Characteristics of NPC patients involved in dysphagia association analysis. **Table S3.** Characteristics of NPC patients involved in oral mucositis association analysis. **Table S4.** Characteristics of NPC patients involved in salivary glands toxicity association analysis. **Table S5.** Characteristics of NPC patients involved in myelosuppression association analysis. **Table S6.** Stratified analysis of the association between skin reaction and chromosome 2q14.2 loci. **Table S7.** Association between therapeutic response and chromosome 2q14.2 loci in the stratified patients.

## Data Availability

The datasets supporting the conclusions of this article are included within the article and its additional files.

## Abbreviations

NPC: Nasopharyngeal carcinoma
GWAS: Genome-wide association study
IMRT: Intensity modulated radiation therapy
GTV: Gross tumor volume
CTV: Clinical target volume
PTV: Planning target volume
RTOG/EORTC: European Organization for Research and Treatment of Cancer
SNPs: Single nucleotide polymorphisms
MAF: Minor Allele Frequency
HWE: Hardy weinberg equilibrium
LD: Linkage disequilibrium
eQTL: Expression quantitative trait Loci
INHBB: Inhibin subunit beta B
TFCP2L1: Transcription factor CP2 like 1
PTPN4: Protein tyrosine phosphatase non-receptor type 4
SYT8: Synaptotagmin 8
EBV: Epstein-barr virus
NBN: Nibrin
TGF-β: Transforming growth factor-beta
Smad: SMAD family member 2
IκBα: NF-κB inhibitor alpha
NF-κB: Nuclear factor kappa B
STAT3: Signal transducer and activator of transcription 3
KLF4: KLF transcription factor 4
RHOF: Ras homolog family member F
FOXO: Forkhead box O
SIRT1: Sirtuin 1
RECIST: Response evaluation criteria in solid tumors
CR: Complete response
PR: Partial response
SD: Stable disease
PD: Progressive disease
PCA: Principal component analysis
BMI: Body mass index
AUC: Area under curve
ROC: Receiver operating characteristic
