# Supplementary material for: A two-stage genome-wide association study to identify novel genetic loci associated with acute radiotherapy toxicity in nasopharyngeal carcinoma
Source: Mol Cancer. 2022 Aug 23;21:169. doi: 10.1186/s12943-022-01631-8 (PMC9400233; doi:10.1186/s12943-022-01631-8)
Supplement: Supplementary file 1 — Additional file 1: Fig. S1. Diagram of data processing flow. Bioinformatics tools utilized in each step were showed in blue in the brackets. Detailed parameters and quality control criteria were indicated with red. Fig. S2. Distribution of samples according to PCA analysis in discovery stage. The red and green spots represented two different groups of patients. The results showed that no stray samples appeared in all five toxicities. Fig. S3. Quantile–quantile (QQ) plot of observed association P values (y-axis) against expected P values (x-axis) in the discovery stage. Fig. S4. Establishment of prediction models for skin reaction (A and B) and dysphagia toxicities (C and D). For each toxicity, patients were firstly randomly divided into two groups, which used to establish (A and C) and test models (B and D) respectively. Then, three multivariable logistic regression models with genetic factors only, clinical factors only and combination of both genetic and clinical factors were established. The genetic model only involved genetic factors: rs6711678, rs4848597, rs4848598 and rs2091255 for skin reaction, and rs584547 for dysphagia. During the calculation, rs6711678, rs4848597, rs4848598 and rs2091255 were combined as polygenic risk scores. The clinical model involved clinical factors only, which include age, sex, BMI, smoking status, stage, EBV infection and radiotherapeutic regimen. The combined model integrated both genetic and clinical factors. BMI: body mass index, EBV: Epstein-Barr virus, AUC: area under curve. Fig. S5. The MAF of rs6711678, rs4848597, rs4848598, rs2091255 and rs584547 in different ethnic populations. AFR: African, EAS: East Asian, EUR: Europe, AMR: American, SAS: South Asian, LAM: Latin American. Table S1. Characteristics of NPC patients involved in skin reaction association analysis. Table S2. Characteristics of NPC patients involved in dysphagia association analysis. Table S3. Characteristics of NPC patients involved in oral mucositis associat [file 12943_2022_1631_MOESM1_ESM.zip › Additional file 1.docx]

**Figure Legends for Supplementary Figures:**

**Fig S1. Diagram of data processing flow.** Bioinformatics tools utilized in each step were showed in blue in the brackets. Detailed parameters and quality control criteria were indicated with red.

**Fig S2. Distribution of samples according to PCA analysis in discovery stage.** The red and green spots represented two different groups of patients. The results showed that no stray samples appeared in all five toxicities.

**Fig S3. Quantile–quantile (QQ) plot of observed association P values (y-axis) against expected P values (x-axis) in the discovery stage.**

**Fig S4. Establishment of prediction models for skin reaction (A and B) and dysphagia toxicities (C and D).** For each toxicity, patients were firstly randomly divided into two groups, which used to establish (A and C) and test models (B and D) respectively. Then, three multivariable logistic regression models with genetic factors only, clinical factors only and combination of both genetic and clinical factors were established. The genetic model only involved genetic factors: rs6711678, rs4848597, rs4848598 and rs2091255 for skin reaction, and rs584547 for dysphagia. During the calculation, rs6711678, rs4848597, rs4848598 and rs2091255 were combined as polygenic risk scores. The clinical model involved clinical factors only, which include age, sex, BMI, smoking status, stage, EBV infection and radiotherapeutic regimen. The combined model integrated both genetic and clinical factors. BMI: body mass index, EBV: Epstein-Barr virus, AUC: area under curve.

**Fig S5. The MAF of rs6711678, rs4848597, rs4848598, rs2091255 and** **rs584547 in different ethnic populations.** AFR: African, EAS: East Asian, EUR: Europe, AMR: American, SAS: South Asian, LAM: Latin American.

**Table S1 Characteristics of NPC patients involved in skin reaction association analysis**

| Characteristics | Discovery stage | | | Validation stage | | | Combined | | |
| --- | --- | --- | --- | --- | --- | --- | --- | --- | --- |
|  | Grade 0 and 1 (n=221) | Grade 2 and 3 (n=94) | P value | Grade 0 and 1 (n=397) | Grade 2 and 3 (n=163) | P value | Grade 0 and 1 (n=618) | Grade 2 and 3 (n=257) | P value |
| Age (Mean ± SD) | 51.63 ± 12.25 | 48.89 ± 11.15 | 0.064 | 48.00 ± 9.25 | 48.08 ± 10.30 | 0.929 | 49.33 ± 10.55 | 48.25 ± 10.57 | 0.171 |
| Gender | | | | | | | | | |
| Male | 143 | 69 | 0.132 | 286 | 127 | 0.151 | 429 | 196 | 0.041 |
| Female | 78 | 25 |  | 111 | 36 |  | 189 | 61 |  |
| BMI (Mean ± SD) | 22.13 ± 2.97 | 23.63 ± 4.02 | <0.001 | 23.46 ± 3.38 | 23.03 ± 3.23 | 0.171 | 22.99 ± 3.29 | 22.94 ± 3.42 | 0.34 |
| Smoking status | | | | | | | | | |
| Nonsmoker | 123 | 43 | 0.107 | 212 | 71 | 0.058 | 335 | 114 | 0.013 |
| Smoker | 98 | 51 |  | 177 | 85 |  | 275 | 136 |  |
| NR | 0 | 0 |  | 8 | 7 |  | 8 | 7 |  |
| EBV | | | | | | | | | |
| Positive | 148 | 64 | 0.935 | 211 | 86 | 0.987 | 359 | 150 | 0.997 |
| Negative | 72 | 30 |  | 163 | 67 |  | 235 | 97 |  |
| NR | 1 | 0 |  | 23 | 10 |  | 24 | 10 |  |
| Clinical stage | | | | | | | | | |
| I | 7 | 0 | 0.055 | 11 | 3 | 0.203 | 18 | 3 | 0.304 |
| II | 14 | 12 |  | 40 | 15 |  | 54 | 27 |  |
| III | 80 | 39 |  | 184 | 65 |  | 264 | 104 |  |
| IV | 119 | 43 |  | 140 | 74 |  | 259 | 117 |  |
| NR | 1 | 0 |  | 22 | 6 |  | 23 | 6 |  |
| Treatment scheme | | | | | | | | | |
| RT alone | 50 | 10 | 0.001 | 6 | 5 | 0.268 | 56 | 15 | 0.090 |
| RT+IC/AC | 4 | 0 |  | 22 | 7 |  | 26 | 7 |  |
| CCRT | 72 | 27 |  | 30 | 7 |  | 102 | 34 |  |
| CCRT+IC/AC | 71 | 57 |  | 328 | 136 |  | 399 | 193 |  |
| RT+other treatment* | 2 | 0 |  | 11 | 8 |  | 13 | 8 |  |

**Table S2 Characteristics of NPC patients involved in dysphagia association analysis**

| Characteristics | Discovery stage | | | Validation stage | | | Combined | | |
| --- | --- | --- | --- | --- | --- | --- | --- | --- | --- |
|  | Grade 0 and 1 (n=146) | Grade 2 and 3 (n=72) | P value | Grade 0 and 1 (n=42) | Grade 2 and 3 (n=68) | P value | Grade 0 and 1 (n=188) | Grade 2 and 3 (n=140) | P value |
| Age (Mean ± SD) | 51.63 ± 12.35 | 50.29 ± 11.12 | 0.440 | 45.67 ± 11.63 | 48.16 ± 9.90 | 0.237 | 50.33 ± 12.44 | 49.26 ± 10.60 | 0.427 |
| Gender | | | | | | | | | |
| Male | 98 | 49 |  | 33 | 47 | 0.279 | 131 | 96 | 0.830 |
| Female | 48 | 23 |  | 9 | 21 |  | 57 | 44 |  |
| BMI (Mean ± SD) | 22.72 ± 3.71 | 22.47 ± 3.35 | 0.632 | 23.88 ± 3.07 | 23.55 ± 3.77 | 0.632 | 22.95 ± 3.61 | 22.99 ± 3.60 |  |
| Smoking status | | | | | | | | | |
| Nonsmoker | 76 | 37 | 0.926 | 21 | 33 | 0.881 | 97 | 70 | 0.775 |
| Smoker | 70 | 35 |  | 21 | 35 |  | 91 | 70 |  |
| NR | 0 | 0 |  | 0 | 0 |  | 0 | 0 |  |
| EBV | | | | | | | | | |
| Positive | 101 | 51 | 0.858 | 20 | 34 | 0.502 | 121 | 85 | 0.140 |
| Negative | 44 | 21 |  | 19 | 25 |  | 63 | 46 |  |
| NR | 1 | 0 |  | 3 | 9 |  | 4 | 9 |  |
| Clinical stage | | | | | | | | | |
| I | 2 | 3 | 0.601 | 2 | 0 | 0.192 | 4 | 3 | 0.950 |
| II | 10 | 6 |  | 5 | 4 |  | 15 | 10 |  |
| III | 54 | 25 |  | 19 | 34 |  | 73 | 59 |  |
| IV | 79 | 38 |  | 16 | 30 |  | 95 | 68 |  |
| NR | 1 | 0 |  | 0 | 0 |  | 1 | 0 |  |
| Treatment scheme | | | | | | | | | |
| RT alone | 26 | 15 | 0.612 | 2 | 0 | 0.349 | 28 | 15 | 0.075 |
| RT+IC/AC | 3 | 0 |  | 3 | 3 |  | 6 | 3 |  |
| CCRT | 47 | 20 |  | 3 | 3 |  | 50 | 23 |  |
| CCRT+IC/AC | 69 | 37 |  | 33 | 61 |  | 102 | 98 |  |
| RT+other treatment* | 1 | 0 |  | 1 | 1 |  | 2 | 1 |  |

**Table S3 Characteristics of NPC patients involved in oral mucositis association analysis**

| Characteristics | Discovery stage | | | Validation stage | | | Combined | | |
| --- | --- | --- | --- | --- | --- | --- | --- | --- | --- |
|  | Grade 0 and 1 (n=83) | Grade 2 and 3 (n=234) | P value | Grade 0 and 1 (n=271) | Grade 2 and 3 (n=354) | P value | Grade 0 and 1 (n=354) | Grade 2 and 3 (n=588) | P value |
| Age (Mean ± SD) | 52.89 ± 11.45 | 50.07 ± 12.05 | 0.061 | 48.24 ± 10.00 | 47.96 ± 9.07 | 0.719 | 49.28 ± 10.56 | 48.76 ± 10.34 | 0.461 |
| Gender | | | | | | | | | |
| Male | 57 | 157 | 0.792 | 197 | 265 | 0.541 | 254 | 422 | 0.995 |
| Female | 26 | 77 |  | 74 | 89 |  | 100 | 166 |  |
| BMI (Mean ± SD) | 22.85 ± 3.14 | 22.50 ± 3.49 | 0.433 | 23.29 ± 3.29 | 23.23 ± 3.35 | 0.830 | 23.20 ± 3.24 | 22.94 ± 3.42 | 0.257 |
| Smoking status | | | | | | | | | |
| Nonsmoker | 41 | 125 | 0.529 | 135 | 182 | 0.616 | 176 | 307 | 0.434 |
| Smoker | 42 | 109 |  | 132 | 164 |  | 174 | 273 |  |
| NR | 0 | 0 |  | 4 | 8 |  | 4 | 8 |  |
| EBV | | | | | | | | | |
| Positive | 47 | 166 | 0.020 | 144 | 188 | 0.420 | 191 | 354 | 0.108 |
| Negative | 35 | 67 |  | 117 | 145 |  | 152 | 212 |  |
| NR | 1 | 1 |  | 10 | 21 |  | 11 | 22 |  |
| Clinical stage | | | | | | | | | |
| I | 2 | 5 | 0.509 | 9 | 7 | 0.073 | 11 | 12 | 0.056 |
| II | 10 | 16 |  | 19 | 35 |  | 29 | 51 |  |
| III | 31 | 88 |  | 140 | 154 |  | 171 | 242 |  |
| IV | 40 | 124 |  | 93 | 148 |  | 133 | 272 |  |
| NR | 0 | 1 |  | 10 | 10 |  | 10 | 11 |  |
| Treatment scheme | | | | | | | | | |
| RT alone | 15 | 45 | 0.681 | 5 | 7 | 0.700 | 20 | 52 | 0.077 |
| RT+IC/AC | 0 | 4 |  | 19 | 16 |  | 19 | 20 |  |
| CCRT | 28 | 71 |  | 10 | 12 |  | 38 | 83 |  |
| CCRT+IC/AC | 39 | 113 |  | 230 | 312 |  | 269 | 425 |  |
| RT+other treatment* | 1 | 1 |  | 7 | 7 |  | 8 | 8 |  |

**Table S4 Characteristics of NPC patients involved in salivary glands toxicity association analysis**

| Characteristics | Discovery stage | | | Validation stage | | | Combined | | |
| --- | --- | --- | --- | --- | --- | --- | --- | --- | --- |
|  | Grade 0 and 1 (n=239) | Grade 2 and 3 (n=67) | P value | Grade 0 and 1 (n=273) | Grade 2 and 3 (n=24) | P value | Grade 0 and 1 (n=512) | Grade 2 and 3 (n=91) | P value |
| Age (Mean ± SD) | 50.13 ± 12.03 | 53.15 ± 11.40 | 0.068 | 47.50 ± 9.63 | 50.04 ± 10.68 | 0.222 | 48.73 ± 10.90 | 52.33 ± 11.30 | 0.004 |
| Gender | | | | | | | | | |
| Male | 161 | 42 | 0.474 | 197 | 21 | 0.103 | 358 | 63 | 0.895 |
| Female | 78 | 25 |  | 76 | 3 |  | 154 | 28 |  |
| BMI (Mean ± SD) | 22.65 ± 3.52 | 22.52 ± 2.93 | 0.791 | 23.55 ± 3.19 | 23.72 ± 4.11 | 0.810 | 23.13 ± 3.37 | 22.84 ± 3.33 | 0.449 |
| Smoking status | | | | | | | | | |
| Nonsmoker | 126 | 37 | 0.741 | 139 | 13 | 0.815 | 265 | 50 | 0.638 |
| Smoker | 112 | 30 |  | 130 | 11 |  | 242 | 41 |  |
| NR | 0 | 0 |  | 4 | 0 |  | 4 | 0 |  |
| EBV | | | | | | | | | |
| Positive | 152 | 51 | 0.072 | 145 | 13 | 0.943 | 297 | 64 | 0.074 |
| Negative | 86 | 16 |  | 112 | 10 |  | 198 | 26 |  |
| NR | 0 | 0 |  | 16 | 1 |  | 16 | 1 |  |
| Clinical stage | | | | | | | | | |
| I | 7 | 0 | 0.100 | 6 | 0 | 0.380 | 13 | 0 | 0.073 |
| II | 19 | 6 |  | 24 | 0 |  | 43 | 6 |  |
| III | 98 | 19 |  | 132 | 14 |  | 230 | 33 |  |
| IV | 113 | 41 |  | 106 | 10 |  | 219 | 51 |  |
| NR | 1 | 1 |  | 5 | 0 |  | 6 | 1 |  |
| Treatment scheme | | | | | | | | | |
| RT alone | 46 | 11 | 0.306 | 4 | 2 | 0.170 | 50 | 13 | 0.320 |
| RT+IC/AC | 6 | 0 |  | 14 | 1 |  | 20 | 1 |  |
| CCRT | 78 | 18 |  | 10 | 0 |  | 88 | 18 |  |
| CCRT+IC/AC | 105 | 38 |  | 241 | 21 |  | 346 | 59 |  |
| RT+other treatment* | 2 | 0 |  | 4 | 0 |  | 6 | 0 |  |

**Table S5 Characteristics of NPC patients involved in myelosuppression association analysis**

| Characteristics | Discovery stage | | |
| --- | --- | --- | --- |
|  | Grade = 0 (%) (n=99) | Grade ≥ 1 (%) (n=219) | P value |
| Age (Mean ± SD) | 52.69 ± 11.47 | 49.89 ± 12.09 | 0.054 |
| Gender | | | |
| Male | 62 | 153 | 0.202 |
| Female | 37 | 66 |  |
| BMI (Mean ± SD) | 22.85 ± 3.71 | 22.50 ± 3.25 | 0.395 |
| Smoking status | | | |
| Nonsmoker | 46 | 121 | 0.146 |
| Smoker | 53 | 98 |  |
| NR | 0 | 0 |  |
| EBV | | | |
| Positive | 58 | 156 | 0.019 |
| Negative | 41 | 61 |  |
| NR | 0 | 2 |  |
| Clinical stage | | | |
| I | 4 | 3 | 0.004 |
| II | 15 | 11 |  |
| III | 38 | 81 |  |
| IV | 42 | 123 |  |
| NR | 0 | 1 |  |
| Treatment scheme | | | |
| RT alone | 38 | 22 | <0.001 |
| RT+IC/AC | 4 | 2 |  |
| CCRT | 30 | 69 |  |
| CCRT+IC/AC | 27 | 124 |  |
| RT+other treatment* | 0 | 2 |  |

**Table S6 Stratified analysis of the association between skin reaction and chromosome 2q14.2 loci**

| Characteristics | rs6711678 | | rs4848597 | | rs4848598 | | rs2091255 | |
| --- | --- | --- | --- | --- | --- | --- | --- | --- |
|  | P value | OR (95%CI) | P value | OR (95%CI) | P value | OR (95%CI) | P value | OR (95%CI) |
| Smoking status |  |  |  |  |  |  |  |  |
| Nonsmoker | 0.028 | 1.460 (1.041-2.047) | 0.023 | 1.484 (1.057-2.085) | 0.032 | 1.451 (1.033-2.037) | 0.017 | 1.513 (1.078-2.122) |
| Smoker | 0.024 | 1.524 (1.058-2.195) | 0.042 | 1.457 (1.014-2.093) | 0.016 | 1.573 (1.090-2.271) | 0.014 | 1.586 (1.097-2.292) |
| EBV |  |  |  |  |  |  |  |  |
| Positive | 0.045 | 1.503 (1.009-2.239) | 0.031 | 1.547 (1.040-2.301) | 0.017 | 1.633 (1.090-2.447) | 0.020 | 1.616 (1.078-2.423) |
| Negative | 0.133 | 1.369 (0.909-2.062) | 0.125 | 1.378 (0.915-2.075) | 0.166 | 1.335 (0.887-2.011) | 0.086 | 1.432 (0.951-2.156) |
| Clinical stage |  |  |  |  |  |  |  |  |
| I+II | 0.829 | 0.912 (0.395-2.104) | 0.829 | 0.912 (0.395-2.104) | 0.829 | 0.912 (0.395-2.104) | 0.829 | 0.912 (0.395-2.104) |
| III+IV | 1.67×10^-4^ | 1.663 (1.276-2.167) | 1.94×10^-4^ | 1.654 (1.270-2.156) | 9.85×10^-5^ | 1.697 (1.301-2.215) | 4.15×10^-5^ | 1.747 (1.338-2.281) |
| Treatment scheme |  |  |  |  |  |  |  |  |
| CCRT | 0.161 | 1.718 (0.806-3.663) | 0.136 | 1.776 (0.835-3.780) | 0.103 | 1.919 (0.876-4.206) | 0.086 | 1.985 (0.907-4.342) |
| CCRT+IC/AC | 2.99×10^-3^ | 1.503 (1.148-1.966) | 4.49×10^-3^ | 1.478 (1.129-1.934) | 3.11×10^-3^ | 1.501 (1.147-1.966) | 1.83×10^-3^ | 1.536 (1.172-2.011) |

**Table S7 Association between therapeutic response and chromosome 2q14.2 loci in the stratified patients**

| SNP | MAF | Additive | | Dominant | | Recessive | |
| --- | --- | --- | --- | --- | --- | --- | --- |
|  |  | P value | OR (95%CI) | P value | OR (95%CI) | P value | OR (95%CI) |
| rs6711678 | 0.30 | 8.09×10^-4^ | 1.96 (1.32-2.90) | 1.10×10^-3^ | 2.66 (1.48-4.78) | 0.04 | 2.18 (1.02-4.67) |
| rs4848597 | 0.31 | 1.16×10^-3^ | 1.92 (1.30-2.86) | 1.88×10^-3^ | 2.54 (1.41-4.56) | 0.04 | 2.19 (1.03-4.69) |
| rs4848598 | 0.30 | 7.02×10^-4^ | 1.98 (1.33-2.94) | 1.20×10^-3^ | 2.64 (1.47-4.74) | 0.03 | 2.28 (1.06-4.89) |
| rs2091255 | 0.29 | 8.32×10^-4^ | 1.96 (1.32-2.92) | 1.88×10^-3^ | 2.51 (1.40-4.48) | 0.03 | 2.40 (1.11-5.15) |
