# Supplementary figures and images for: A two-stage genome-wide association study to identify novel genetic loci associated with acute radiotherapy toxicity in nasopharyngeal carcinoma
Source: Mol Cancer. 2022 Aug 23;21:169. doi: 10.1186/s12943-022-01631-8 (PMC9400233; doi:10.1186/s12943-022-01631-8)

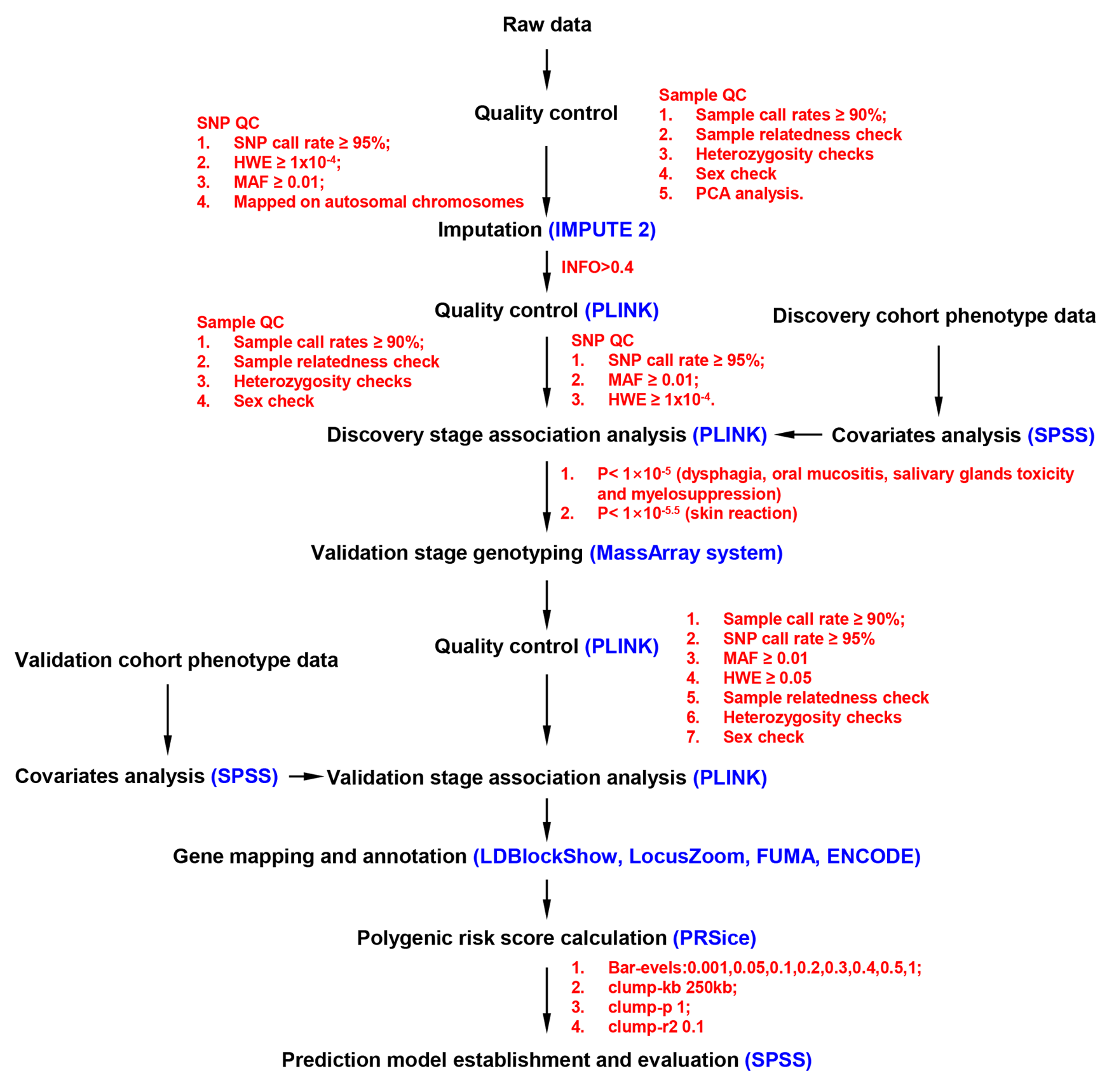

Supplement: Supplementary file 1 — Additional file 1: Fig. S1. Diagram of data processing flow. Bioinformatics tools utilized in each step were showed in blue in the brackets. Detailed parameters and quality control criteria were indicated with red. Fig. S2. Distribution of samples according to PCA analysis in discovery stage. The red and green spots represented two different groups of patients. The results showed that no stray samples appeared in all five toxicities. Fig. S3. Quantile–quantile (QQ) plot of observed association P values (y-axis) against expected P values (x-axis) in the discovery stage. Fig. S4. Establishment of prediction models for skin reaction (A and B) and dysphagia toxicities (C and D). For each toxicity, patients were firstly randomly divided into two groups, which used to establish (A and C) and test models (B and D) respectively. Then, three multivariable logistic regression models with genetic factors only, clinical factors only and combination of both genetic and clinical factors were established. The genetic model only involved genetic factors: rs6711678, rs4848597, rs4848598 and rs2091255 for skin reaction, and rs584547 for dysphagia. During the calculation, rs6711678, rs4848597, rs4848598 and rs2091255 were combined as polygenic risk scores. The clinical model involved clinical factors only, which include age, sex, BMI, smoking status, stage, EBV infection and radiotherapeutic regimen. The combined model integrated both genetic and clinical factors. BMI: body mass index, EBV: Epstein-Barr virus, AUC: area under curve. Fig. S5. The MAF of rs6711678, rs4848597, rs4848598, rs2091255 and rs584547 in different ethnic populations. AFR: African, EAS: East Asian, EUR: Europe, AMR: American, SAS: South Asian, LAM: Latin American. Table S1. Characteristics of NPC patients involved in skin reaction association analysis. Table S2. Characteristics of NPC patients involved in dysphagia association analysis. Table S3. Characteristics of NPC patients involved in oral mucositis associat [file 12943_2022_1631_MOESM1_ESM.zip › Figure S1.tif]

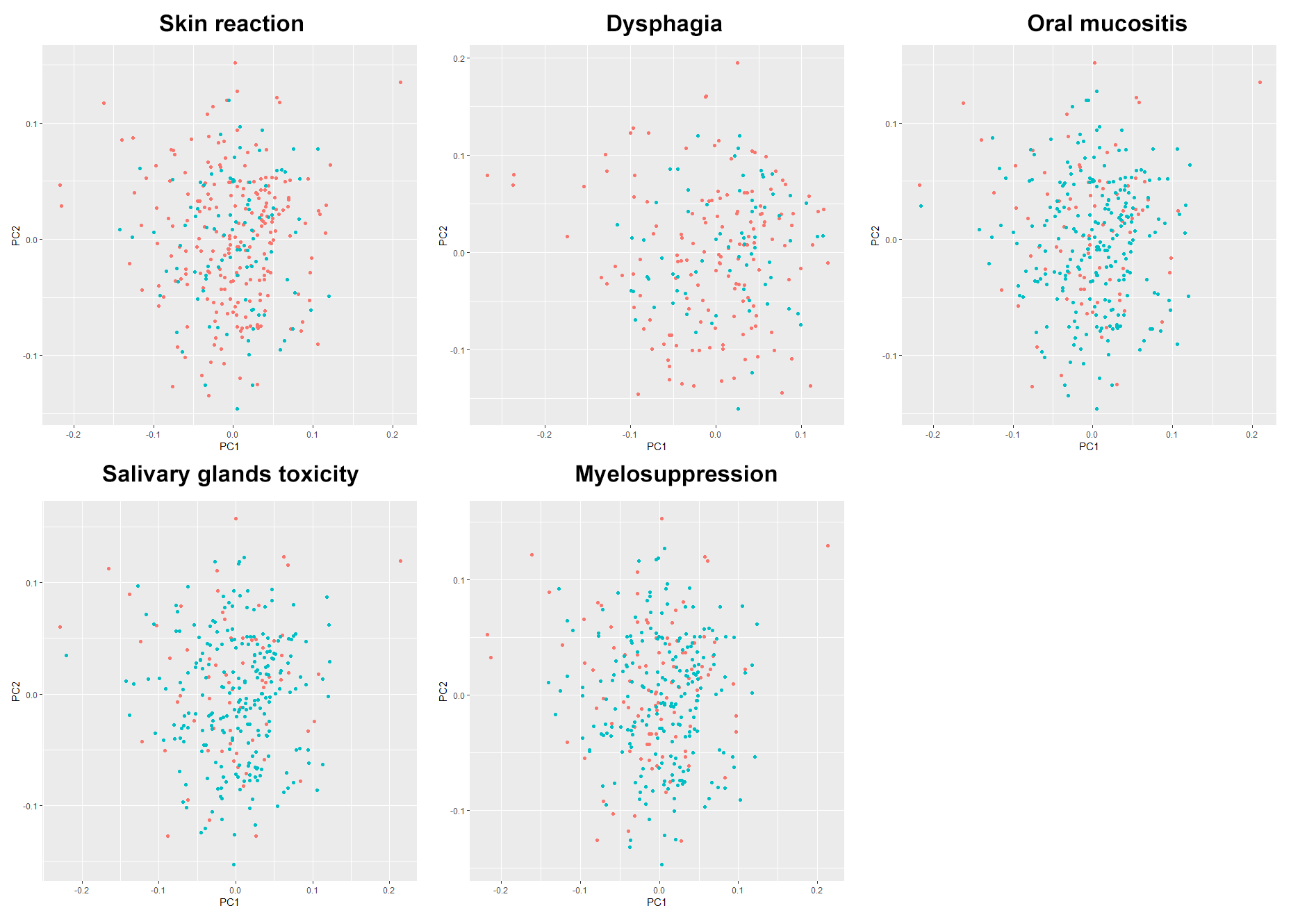

Supplement: Supplementary file 1 — Additional file 1: Fig. S1. Diagram of data processing flow. Bioinformatics tools utilized in each step were showed in blue in the brackets. Detailed parameters and quality control criteria were indicated with red. Fig. S2. Distribution of samples according to PCA analysis in discovery stage. The red and green spots represented two different groups of patients. The results showed that no stray samples appeared in all five toxicities. Fig. S3. Quantile–quantile (QQ) plot of observed association P values (y-axis) against expected P values (x-axis) in the discovery stage. Fig. S4. Establishment of prediction models for skin reaction (A and B) and dysphagia toxicities (C and D). For each toxicity, patients were firstly randomly divided into two groups, which used to establish (A and C) and test models (B and D) respectively. Then, three multivariable logistic regression models with genetic factors only, clinical factors only and combination of both genetic and clinical factors were established. The genetic model only involved genetic factors: rs6711678, rs4848597, rs4848598 and rs2091255 for skin reaction, and rs584547 for dysphagia. During the calculation, rs6711678, rs4848597, rs4848598 and rs2091255 were combined as polygenic risk scores. The clinical model involved clinical factors only, which include age, sex, BMI, smoking status, stage, EBV infection and radiotherapeutic regimen. The combined model integrated both genetic and clinical factors. BMI: body mass index, EBV: Epstein-Barr virus, AUC: area under curve. Fig. S5. The MAF of rs6711678, rs4848597, rs4848598, rs2091255 and rs584547 in different ethnic populations. AFR: African, EAS: East Asian, EUR: Europe, AMR: American, SAS: South Asian, LAM: Latin American. Table S1. Characteristics of NPC patients involved in skin reaction association analysis. Table S2. Characteristics of NPC patients involved in dysphagia association analysis. Table S3. Characteristics of NPC patients involved in oral mucositis associat [file 12943_2022_1631_MOESM1_ESM.zip › Figure S2.tif]

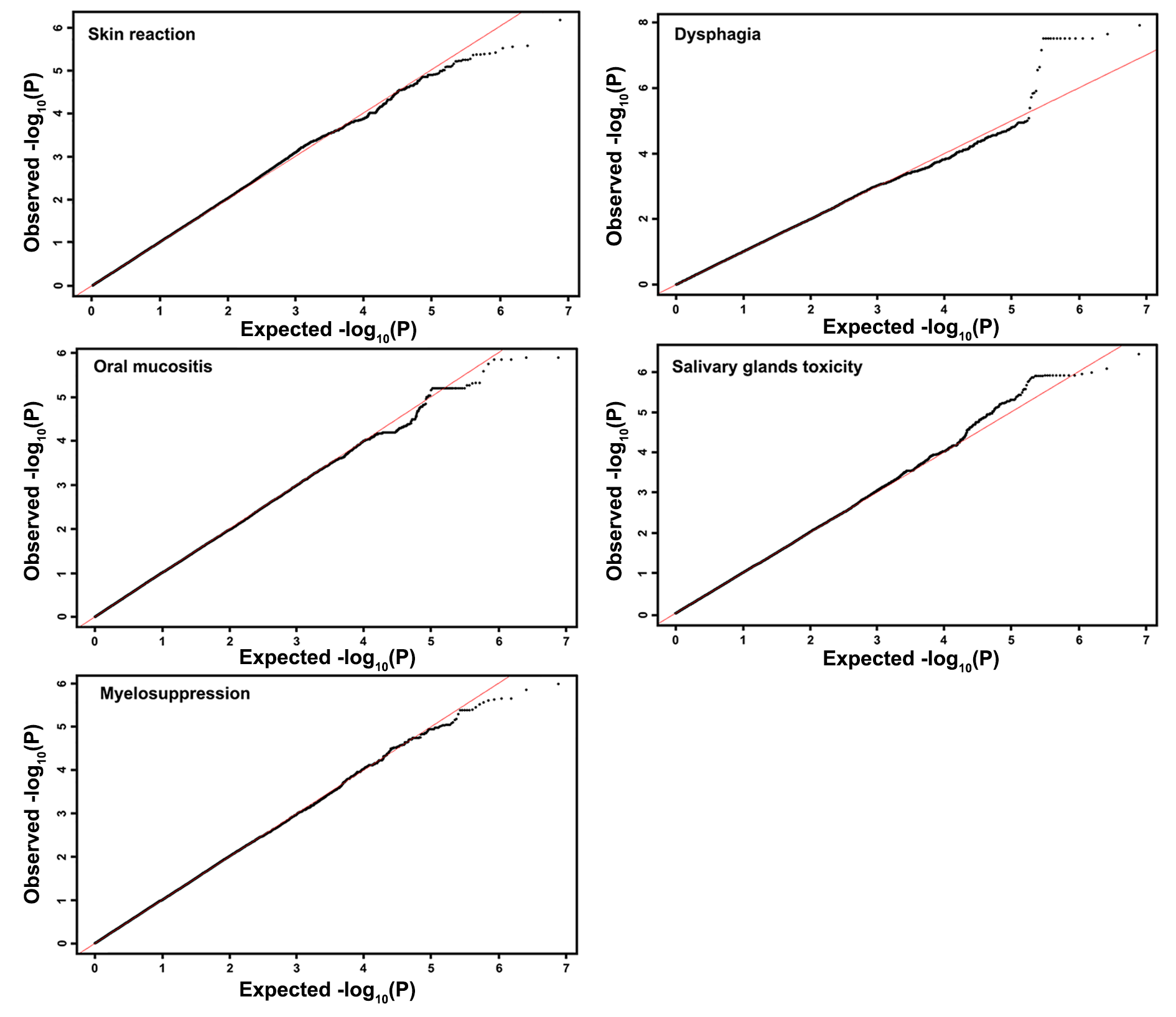

Supplement: Supplementary file 1 — Additional file 1: Fig. S1. Diagram of data processing flow. Bioinformatics tools utilized in each step were showed in blue in the brackets. Detailed parameters and quality control criteria were indicated with red. Fig. S2. Distribution of samples according to PCA analysis in discovery stage. The red and green spots represented two different groups of patients. The results showed that no stray samples appeared in all five toxicities. Fig. S3. Quantile–quantile (QQ) plot of observed association P values (y-axis) against expected P values (x-axis) in the discovery stage. Fig. S4. Establishment of prediction models for skin reaction (A and B) and dysphagia toxicities (C and D). For each toxicity, patients were firstly randomly divided into two groups, which used to establish (A and C) and test models (B and D) respectively. Then, three multivariable logistic regression models with genetic factors only, clinical factors only and combination of both genetic and clinical factors were established. The genetic model only involved genetic factors: rs6711678, rs4848597, rs4848598 and rs2091255 for skin reaction, and rs584547 for dysphagia. During the calculation, rs6711678, rs4848597, rs4848598 and rs2091255 were combined as polygenic risk scores. The clinical model involved clinical factors only, which include age, sex, BMI, smoking status, stage, EBV infection and radiotherapeutic regimen. The combined model integrated both genetic and clinical factors. BMI: body mass index, EBV: Epstein-Barr virus, AUC: area under curve. Fig. S5. The MAF of rs6711678, rs4848597, rs4848598, rs2091255 and rs584547 in different ethnic populations. AFR: African, EAS: East Asian, EUR: Europe, AMR: American, SAS: South Asian, LAM: Latin American. Table S1. Characteristics of NPC patients involved in skin reaction association analysis. Table S2. Characteristics of NPC patients involved in dysphagia association analysis. Table S3. Characteristics of NPC patients involved in oral mucositis associat [file 12943_2022_1631_MOESM1_ESM.zip › Figure S3.tif]

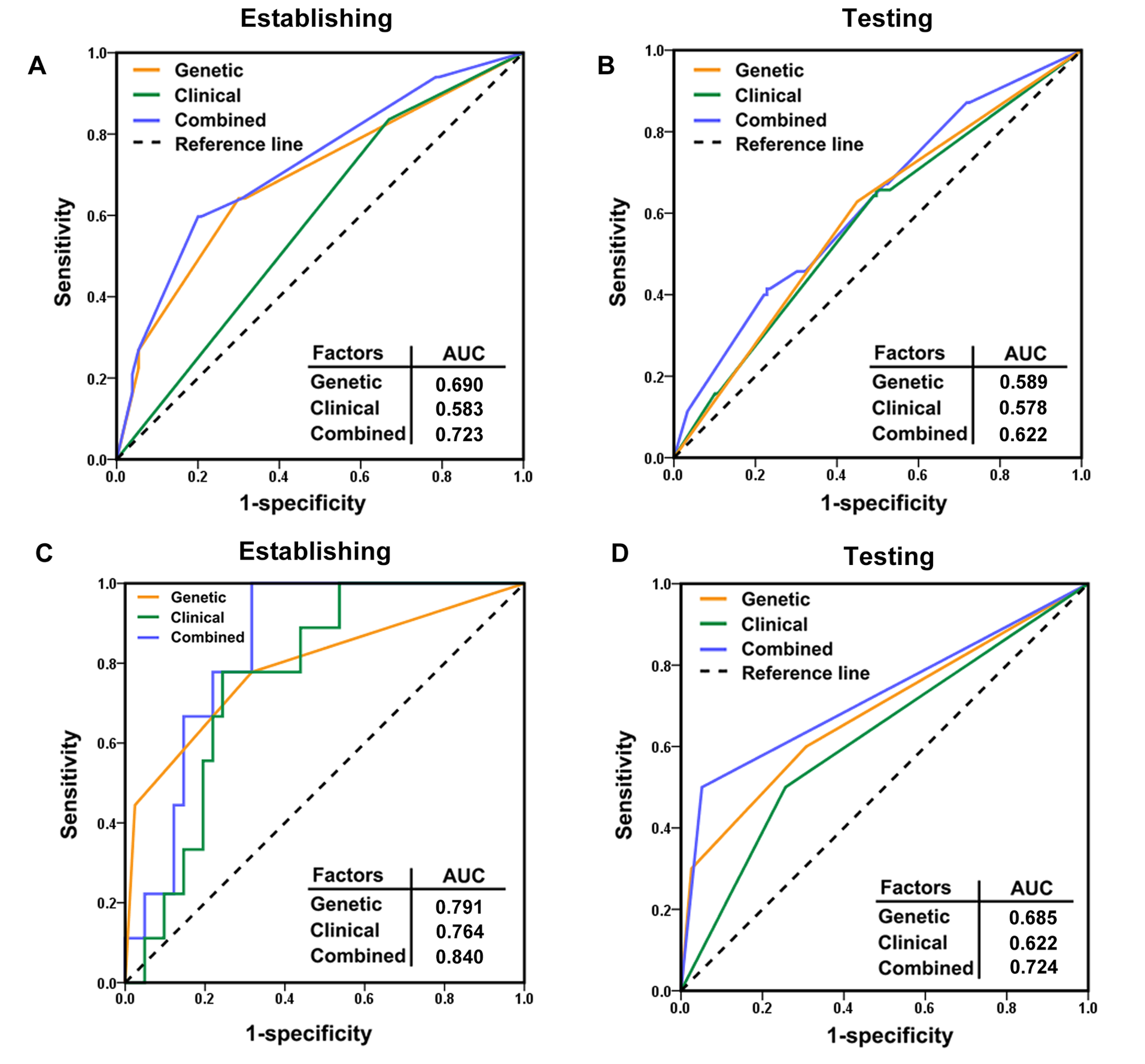

Supplement: Supplementary file 1 — Additional file 1: Fig. S1. Diagram of data processing flow. Bioinformatics tools utilized in each step were showed in blue in the brackets. Detailed parameters and quality control criteria were indicated with red. Fig. S2. Distribution of samples according to PCA analysis in discovery stage. The red and green spots represented two different groups of patients. The results showed that no stray samples appeared in all five toxicities. Fig. S3. Quantile–quantile (QQ) plot of observed association P values (y-axis) against expected P values (x-axis) in the discovery stage. Fig. S4. Establishment of prediction models for skin reaction (A and B) and dysphagia toxicities (C and D). For each toxicity, patients were firstly randomly divided into two groups, which used to establish (A and C) and test models (B and D) respectively. Then, three multivariable logistic regression models with genetic factors only, clinical factors only and combination of both genetic and clinical factors were established. The genetic model only involved genetic factors: rs6711678, rs4848597, rs4848598 and rs2091255 for skin reaction, and rs584547 for dysphagia. During the calculation, rs6711678, rs4848597, rs4848598 and rs2091255 were combined as polygenic risk scores. The clinical model involved clinical factors only, which include age, sex, BMI, smoking status, stage, EBV infection and radiotherapeutic regimen. The combined model integrated both genetic and clinical factors. BMI: body mass index, EBV: Epstein-Barr virus, AUC: area under curve. Fig. S5. The MAF of rs6711678, rs4848597, rs4848598, rs2091255 and rs584547 in different ethnic populations. AFR: African, EAS: East Asian, EUR: Europe, AMR: American, SAS: South Asian, LAM: Latin American. Table S1. Characteristics of NPC patients involved in skin reaction association analysis. Table S2. Characteristics of NPC patients involved in dysphagia association analysis. Table S3. Characteristics of NPC patients involved in oral mucositis associat [file 12943_2022_1631_MOESM1_ESM.zip › Figure S4.tif]

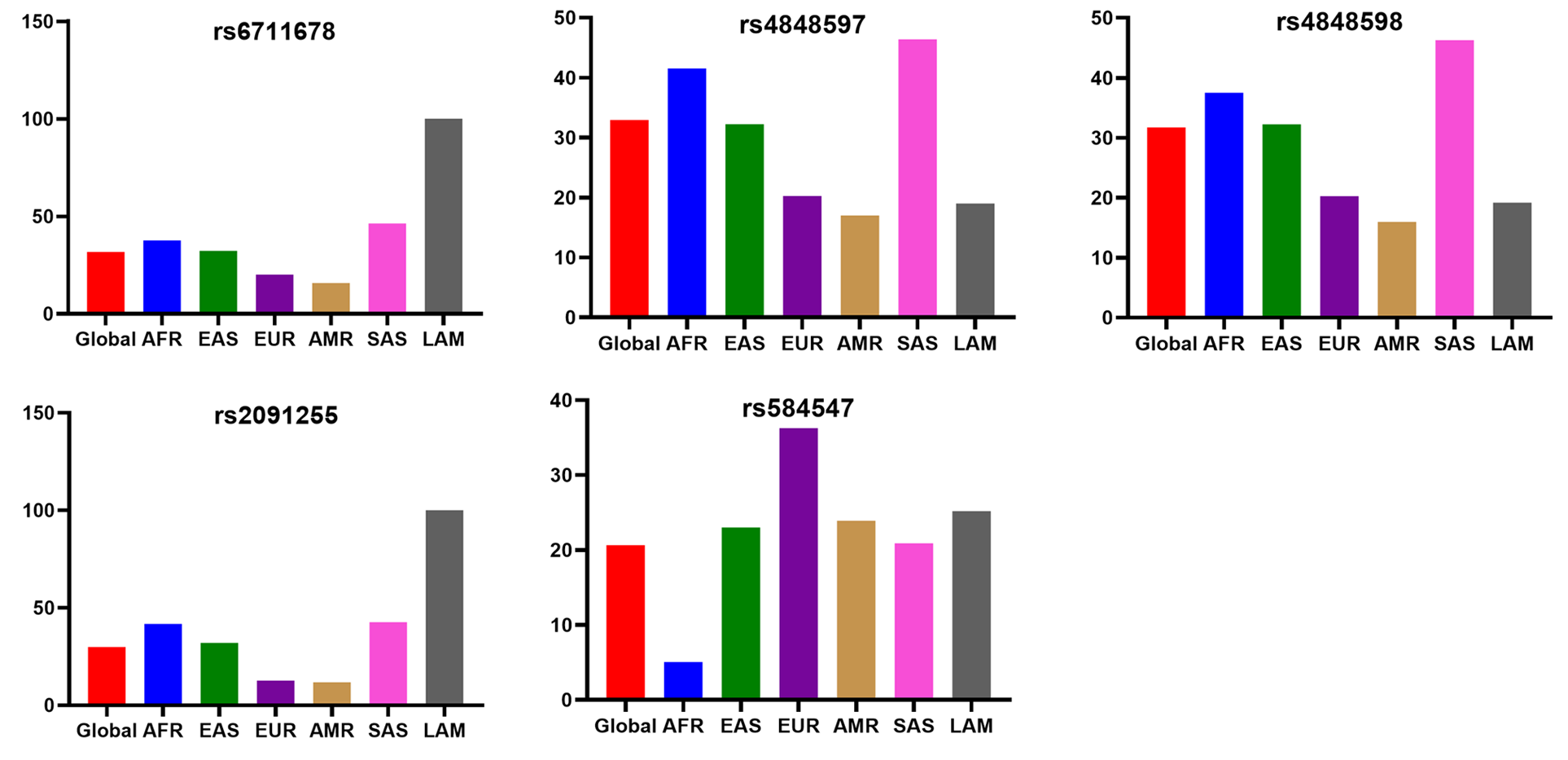

Supplement: Supplementary file 1 — Additional file 1: Fig. S1. Diagram of data processing flow. Bioinformatics tools utilized in each step were showed in blue in the brackets. Detailed parameters and quality control criteria were indicated with red. Fig. S2. Distribution of samples according to PCA analysis in discovery stage. The red and green spots represented two different groups of patients. The results showed that no stray samples appeared in all five toxicities. Fig. S3. Quantile–quantile (QQ) plot of observed association P values (y-axis) against expected P values (x-axis) in the discovery stage. Fig. S4. Establishment of prediction models for skin reaction (A and B) and dysphagia toxicities (C and D). For each toxicity, patients were firstly randomly divided into two groups, which used to establish (A and C) and test models (B and D) respectively. Then, three multivariable logistic regression models with genetic factors only, clinical factors only and combination of both genetic and clinical factors were established. The genetic model only involved genetic factors: rs6711678, rs4848597, rs4848598 and rs2091255 for skin reaction, and rs584547 for dysphagia. During the calculation, rs6711678, rs4848597, rs4848598 and rs2091255 were combined as polygenic risk scores. The clinical model involved clinical factors only, which include age, sex, BMI, smoking status, stage, EBV infection and radiotherapeutic regimen. The combined model integrated both genetic and clinical factors. BMI: body mass index, EBV: Epstein-Barr virus, AUC: area under curve. Fig. S5. The MAF of rs6711678, rs4848597, rs4848598, rs2091255 and rs584547 in different ethnic populations. AFR: African, EAS: East Asian, EUR: Europe, AMR: American, SAS: South Asian, LAM: Latin American. Table S1. Characteristics of NPC patients involved in skin reaction association analysis. Table S2. Characteristics of NPC patients involved in dysphagia association analysis. Table S3. Characteristics of NPC patients involved in oral mucositis associat [file 12943_2022_1631_MOESM1_ESM.zip › Figure S5.tif]
